# Severe Infection Outcomes With SGLT2 Versus DPP‐4 Inhibitors in Type 2 Diabetes: A Systematic Review and Active‐Comparator Meta‐Analysis of Real‐World Cohorts

**DOI:** 10.1002/edm2.70381

**Published:** 2026-11-01

**Authors:** Yung‐Ming Huang, Jen‐Hung Wang, Wan‐Ting Huang, Ji‐Ze Hsu, Soon‐Hian Teh

**Affiliations:** ^1^ Department of Pharmacy Hualien Tzu Chi Hospital, Buddhist Tzu Chi Medical Foundation Hualien City Taiwan; ^2^ Center for Clinical Epidemiology and Biostatistics Hualien Tzu Chi Hospital, Buddhist Tzu Chi Medical Foundation Hualien City Taiwan; ^3^ Division of Infectious Diseases, Department of Internal Medicine Hualien Tzu Chi Hospital, Buddhist Tzu Chi Medical Foundation Hualien City Taiwan; ^4^ Tzu Chi University Hualien City Taiwan

**Keywords:** pneumonia, sepsis, sodium‐glucose transporter 2 inhibitors

## Abstract

**Introduction:**

To update and methodologically refine the synthesis of real‐world evidence on severe infection outcomes with sodium‐glucose cotransporter 2 inhibitors (SGLT2i) versus dipeptidyl peptidase‐4 inhibitors (DPP‐4i), emphasising limits of causal interpretation.

**Methods:**

PubMed, Embase, Scopus and CENTRAL were searched from inception through 29 June 2026. Quantitative synthesis was restricted to DPP‐4i active‐comparator estimates. Incident pneumonia and sepsis were prespecified co‐primary outcomes. Hazard ratios and incidence rate ratios were pooled as related but non‐identical estimands using restricted maximum likelihood with modified Hartung‐Knapp inference, with leave‐one‐out, prediction‐interval and effect‐measure‐stratified analyses. ROBINS‐I and GRADE were applied.

**Results:**

Eighteen cohorts were included; the 11 contributing quantitatively were all at moderate risk of bias. For incident pneumonia (five studies), the pooled ratio was 0.65 (95% CI: 0.55–0.75; prediction interval 0.45–0.93; *I* ^2^ = 89.8%; very low certainty); omitting one multinational cohort whose follow‐up began 2 years after drug initiation yielded 0.61 (0.56–0.67; *I* ^2^ = 0%). For incident sepsis (five studies), the prediction interval crossed the null (0.38–1.13); the exploratory pooled ratio was 0.65 (0.53–0.80; *I* ^2^ = 90.6%, and 88.1%–92.4% after every omission; very low certainty). Hazard‐ratio‐only point estimates were similar, although the sepsis estimate was imprecise (95% CI including 1.0). Two cohorts reported identical, unpooled estimates for infection‐related hospitalisation (0.74 each; low certainty). Comparisons with GLP‐1 receptor agonists did not show a consistent lower‐risk pattern across four cohorts, two in selected populations, and randomised‐trial syntheses have not shown a consistent class‐wide preventive effect.

**Conclusions:**

The available evidence did not suggest an increased average risk of severe infection with SGLT2i compared with DPP‐4i. Given low to very low certainty and substantial heterogeneity, the lower pooled estimates should not be interpreted as a preventive effect.

**Review Registration:** PROSPERO: CRD420261390509.

## Graphical abstract

A systematic review of 18 real‐world cohorts found no increased average risk of severe infection with SGLT2 inhibitors versus DPP‐4 inhibitors. Lower pneumonia and sepsis estimates were heterogeneous and of very low certainty; two unpooled hospitalisation estimates were of low certainty. None should be read as infection prevention.

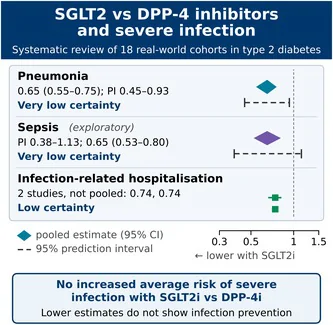

## 1 Introduction

Type 2 diabetes mellitus (T2DM) is associated with increased risks of pneumonia, sepsis, infection‐related hospitalisation and death. Hyperglycaemia, impaired innate immunity, chronic kidney disease, frailty and cardiopulmonary comorbidity all contribute to this excess burden, making severe infection a clinically relevant outcome when glucose‐lowering treatments are compared in routine practice [1, 2, 3, 4].

Sodium‐glucose cotransporter 2 inhibitors (SGLT2i) are now central to the management of T2DM because of their cardiovascular and kidney benefits [5, 6]. Their infection‐related safety profile is usually framed around genital mycotic and urinary tract infection [7]. That framing does not fully address severe infection phenotypes. Several cohorts have reported lower pneumonia, sepsis or infection‐related hospitalisation estimates with SGLT2i, but biological explanations—including reduced inflammation, oxidative stress and cardiorenal decompensation—compete with treatment‐selection explanations such as lower frailty, better vaccination, greater adherence and closer clinical follow‐up among SGLT2i recipients [8, 9].

A recent systematic review of real‐world evidence reported lower estimates for pneumonia, pneumonia‐related mortality and sepsis [10]. Its pneumonia meta‐analysis included three studies, all with DPP‐4i comparators; pneumonia‐related mortality was based on two studies; and its sepsis meta‐analysis included three studies, one of which used a broader non‐SGLT2i comparator. Restricting the comparator to DPP‐4i therefore changes the sepsis contrast more than the pneumonia contrast. Several large active‐comparator cohorts have since been published, including studies of infection‐related hospitalisation, sepsis, advanced kidney disease and post‐discharge populations. These additions increase statistical information but also create methodological challenges: overlapping source populations, different outcome definitions, divergent healthcare settings and residual confounding may produce a precise pooled average that does not describe the range expected across settings.

We therefore conducted an updated and methodologically refined synthesis of this question. Compared with the previous review [10], the principal differences are an expanded evidence base, restriction of the principal quantitative analyses to DPP‐4i active comparators, explicit rules for overlapping data sources, prediction intervals reported alongside confidence intervals, GRADE certainty ratings and consideration of infection‐related hospitalisation as a secondary outcome. Incident pneumonia and incident sepsis were kept as separate co‐primary outcomes. Our aims were to quantify the average associations for severe infection outcomes, determine the extent of between‐study variability and identify the limits of interpretation for clinical practice.

## 2 Materials and Methods

### 2.1 Protocol, Ethics and Reporting

The review was registered in PROSPERO (CRD420261390509) on 19 May 2026, before the final database search, and reported according to PRISMA 2020 and MOOSE [11, 12]. The registration record was updated to reflect the final search date, operational handling of overlapping cohorts and exploratory analyses. Complete search strategies, eligibility decisions, overlap mapping, risk‐of‐bias assessments and sensitivity analyses are provided in the Appendix S1. Reporting‐guideline documentation is described in Section S9.

### 2.2 Search Strategy and Eligibility

PubMed, Embase, Scopus and Cochrane CENTRAL were searched from inception through 29 June 2026. Search terms combined controlled vocabulary and free‐text terms for SGLT2i and individual agents, T2DM and severe infection outcomes; complete strategies are provided in Section S1 and Table S1. No language or publication‐date restrictions were applied.

Eligible studies enrolled adults aged 18 years or older with T2DM, evaluated SGLT2i exposure as a class or individual agent, included a comparator group, and reported at least one prespecified severe infection outcome as an adjusted relative effect estimate with a 95% confidence interval (CI) in a real‐world cohort or nested case–control design. DPP‐4i comparisons were prioritised for quantitative synthesis because both drug classes are commonly used at a similar stage of treatment and therefore offer a clinically meaningful active‐comparator contrast. Studies using other comparators remained eligible for narrative synthesis. We excluded randomised trials, case reports or series, studies limited to Type 1 diabetes, studies reporting only localised genitourinary infection, disproportionality analyses without denominator‐defined cohorts, and conference abstracts superseded by full publications.

### 2.3 Study Selection and Data Extraction

After deduplication, records were managed in Rayyan. Two reviewers independently screened titles and abstracts, assessed full texts and resolved disagreements by consensus. Two reviewers independently extracted study characteristics, jurisdiction and data source, study period, design, exposure and comparator definitions, outcome definitions, follow‐up, adjustment variables and the most fully adjusted effect estimate with its 95% CI. When an abstract and the main results table disagreed, the results table was used. Study authors were not contacted because all required aggregate data were available in published reports and their supplementary materials.

### 2.4 Outcomes and Overlapping Data Sources

We use ‘severe infection outcomes’ as an umbrella term for infection events ascertained from hospital, claims or electronic health records: incident pneumonia, incident sepsis and infection‐related hospitalisation, with ICU admission and infection‐related mortality summarised narratively. These outcomes represent different clinical stages, and not all are systemic infections; each was therefore analysed separately, and none was combined into a composite. Incident pneumonia and incident sepsis were prespecified co‐primary outcomes and were interpreted separately. Because heterogeneity remained substantial and the 95% prediction interval crossed 1.0, the pooled quantitative synthesis for sepsis is interpreted as exploratory; this interpretive label does not alter the prespecified outcome hierarchy. Secondary outcomes were infection‐related hospitalisation, infection‐related mortality, pneumonia‐related mortality, ICU admission and septic shock. Infection‐related hospitalisation required a study‐reported estimate for overall infection‐related admissions; diagnosis‐chapter or organ‐system categories were not accepted as substitutes. Studies of prognosis among patients already hospitalised with pneumonia were not pooled with incident‐pneumonia studies. All‐cause mortality and all‐cause hospitalisation were not accepted as infection endpoints.

Potential overlap was assessed at the data‐source and endpoint levels. When studies used the same or substantially overlapping source population and reported the same endpoint, the primary synthesis retained the estimate most closely aligned with the target contrast, considering use of a DPP‐4i active comparator, incident‐user design, incident severe‐infection endpoint and an unselected T2DM population. Alternative estimates were retained for sensitivity or narrative interpretation. Hong Kong Clinical Data Analysis and Reporting System cohorts, TriNetX federated‐network cohorts and Australian linked‐data cohorts were evaluated explicitly. Ng 2023 reported ICU admission for sepsis, a severity‐conditional endpoint, from the same CDARS new‐user enrolment window (2015–2019) as Wu MZ 2022; it was therefore not pooled with incident sepsis but was used in a substitution sensitivity analysis [13, 14]. Three DPP‐4i‐comparator cohorts were drawn from the same federated network: an unselected multinational Type 2 diabetes cohort, a cohort restricted to end‐stage kidney disease and a cohort restricted to psoriasis. The unselected cohort was retained in the primary synthesis because it was most closely aligned with the target contrast; the two selected‐population cohorts were retained as overlap‐inclusive sensitivity evidence. Where a study reported both a DPP‐4i and a GLP‐1 receptor‐agonist comparison in the same source population, only the DPP‐4i estimate was eligible for pooling; the alternative‐comparator estimate was used solely to examine comparator dependence. The selected‐population cohorts involving psoriasis, end‐stage kidney disease or advanced chronic kidney disease were used only for qualitative triangulation and were not considered directly transportable to the broader T2DM population. Both Australian estimates were retained because patient‐level overlap could not be quantified; each was removed separately in sensitivity analysis. No overlap proportion was imputed from aggregate reports. Endpoint mapping and data‐source overlap are detailed in Section S3 and Tables S3, S4.

### 2.5 Risk of Bias and Certainty of Evidence

Two reviewers independently assessed risk of bias with ROBINS‐I across seven domains and resolved disagreements by consensus [15]. Particular attention was given to active‐comparator new‐user design, temporality, immortal‐time and time‐lag bias, and adjustment for diabetes severity, kidney function, frailty proxies, prior infection, vaccination and cardiopulmonary comorbidity. Large sample size or propensity‐score adjustment alone was not considered sufficient for a low‐risk judgement. Certainty for each outcome was appraised using GRADE [16].

### 2.6 Statistical Analysis

Adjusted hazard ratios and incidence rate ratios were analysed on the natural‐log scale using generic inverse‐variance random‐effects models for the principal incident‐outcome syntheses. These measures were combined as a pragmatic synthesis of related but non‐identical estimands: hazard ratios from time‐to‐first‐event models, and incidence rate ratios from count models of hospitalisation rates, in which repeat admissions may contribute. For rare outcomes with approximately constant event rates the two measures approximate the same relative rate, but they diverge when recurrence, follow‐up duration or the timing of events differ between groups; analyses were therefore also stratified by effect measure (Tables S10, S11). Odds ratios and risk ratios were not included in the principal incident‐outcome pools. Mortality outcomes were not statistically pooled when the contributing reports differed in cause specificity, clinical time origin or effect measure; they were summarised narratively.

For DPP‐4i active‐comparator outcomes with at least three studies, primary inference used restricted maximum likelihood (REML) random‐effects models with the modified Hartung‐Knapp adjustment; outcomes with fewer than three studies were not pooled. The adjusted standard error was constrained to be no smaller than the conventional random‐effects standard error; the formula and implementation details are provided in Section S7 and Table S13. Confidence intervals used a t distribution with k‐1 degrees of freedom. Prediction intervals used a t distribution with k‐2 degrees of freedom and were reported for analyses with at least three studies; they describe dispersion under the fitted random‐effects model rather than directly testing external validity [17]. Between‐study variability was quantified with τ^2^ (REML, with Q‐profile 95% confidence intervals) and with I^2^ calculated from Cochran's Q as (Q–df)/Q. Prespecified sensitivity analyses (Section S6) included leave‐one‐out analyses, overlap‐inclusive analyses and removal or substitution of potentially overlapping data sources, and conventional DerSimonian‐Laird random‐effects estimates with Wald‐type confidence intervals; study weights and influence diagnostics are reported in Table S12. Exploratory funnel plots and Egger tests were reported only for transparency because no endpoint included 10 studies (Section S10, Table S15 and Figure S1). A post hoc assessment grouped cohorts by data‐source architecture and was considered hypothesis‐generating. Analyses were performed in R version 4.3.1 with metafor version 4.6‐0.

## 3 Results

### 3.1 Study Selection and Characteristics

The searches retrieved 479 records (PubMed 171, Embase 136, Scopus 153 and CENTRAL 19). After removal of 77 duplicates, 402 records were screened, 39 full‐text reports were assessed and 21 were excluded (Section S2 and Table S2). Eighteen studies were included in the qualitative synthesis and 11 contributed estimates to quantitative analyses, 10 of them to at least one pooled analysis (Figure 1); one further overlapping cohort (Ng 2023) was used only in a substitution sensitivity analysis added at revision. Studies were published from 2021 to 2026 and drew on claims, insurance, electronic health record (EHR), registry and hospital‐linked data from eight jurisdictions and multinational federated networks (Table 1). The cohorts added by the updated search are described in Section S13.

**FIGURE 1:**
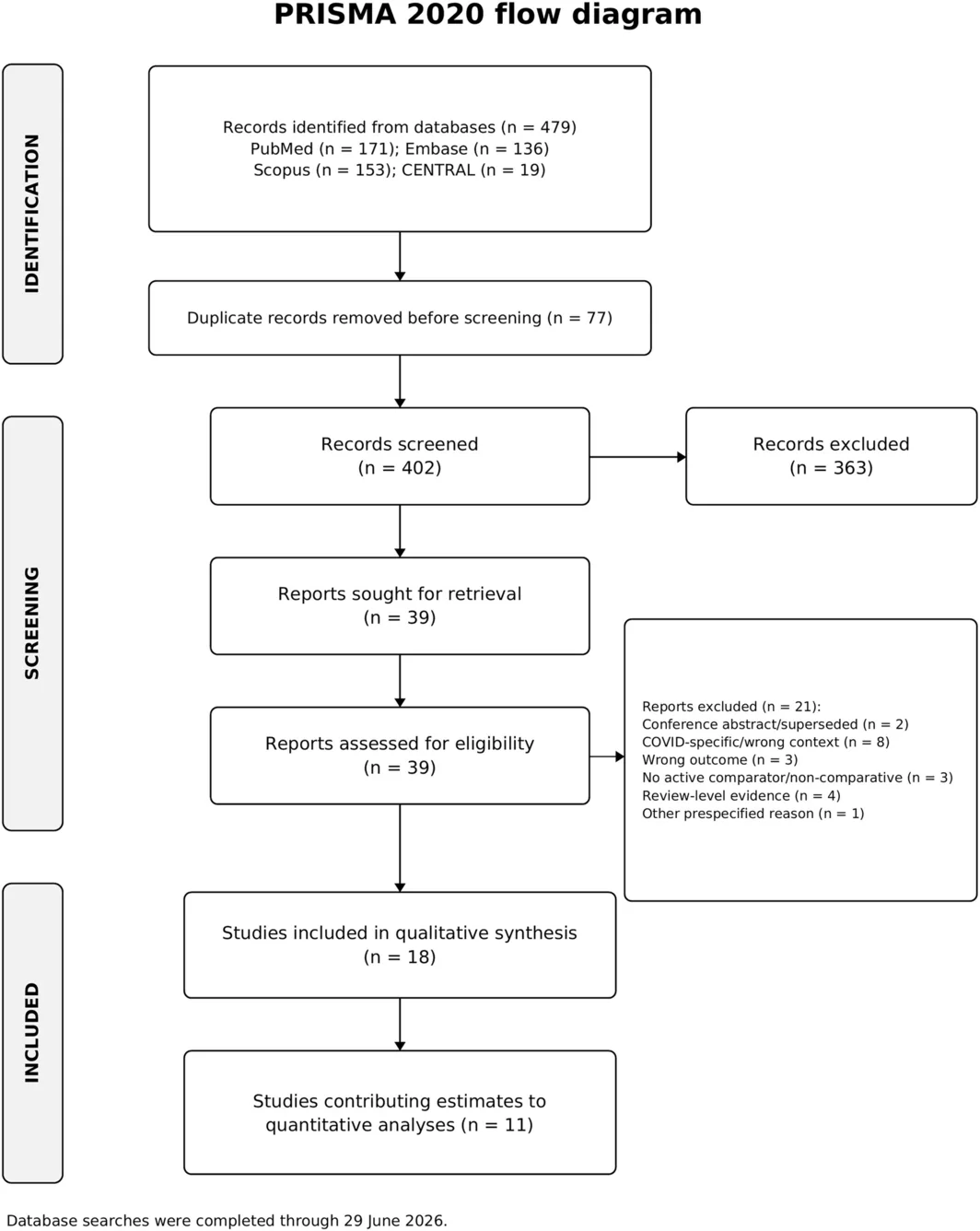
PRISMA 2020 flow diagram for study selection. Database searches were completed through 29 June 2026. Of 479 records, 39 full‐text reports were assessed, 21 were excluded, 18 studies were included in qualitative synthesis and 11 contributed estimates to quantitative analyses, 10 of them to at least one pooled analysis. Ng 2023 contributed only to a substitution sensitivity analysis and is not counted among these.

**TABLE 1:** Characteristics and analytic role of included studies.

Study	Country/data source	Design and comparator	Key outcome(s)	Analytic role/ROBINS‐I
Brunetti et al. (2021) [18]	United Kingdom; CPRD Gold‐HES	Population cohort; time‐dependent exposure; DPP‐4i	Hospitalisation for community‐acquired pneumonia	Primary pneumonia; moderate
Au et al. (2022) [19]	Hong Kong; CDARS	Prevalent new‐user, propensity‐score matched; DPP‐4i	Pneumonia; pneumonia mortality	Sensitivity (CDARS overlap); moderate
Rim et al. (2022) [20]	United States; Veterans Health Administration	Oral antidiabetic cohort; non‐user comparator	Hospital or ICU admission for infection	Narrative; serious
Wu et al. (2022) [13]	Hong Kong; CDARS	New‐user, propensity‐score matched; DPP‐4i	Pneumonia; sepsis; related mortality	Primary pneumonia and sepsis; moderate
Jeong et al. (2023) [21]	South Korea; national claims	Active‐comparator new‐user, propensity‐score matched; DPP‐4i	Respiratory composite; pneumonia	Primary pneumonia; moderate
Ng et al. (2023) [14]	Hong Kong; CDARS	New‐user, propensity‐score matched; DPP‐4i	ICU admission; mortality	Narrative critical illness; sepsis substitution sensitivity; moderate
Narasaki et al. (2024) [22]	United States; Veterans Affairs	Incident‐user; DPP‐4i and GLP‐1 RA	Infection‐related hospitalisation	Hospitalisation (not pooled); moderate
Tan et al. (2024) [23]	Australia; NDSS‐PBS‐hospital‐NDI	Active‐comparator new‐user, outcome‐wide; DPP‐4i	Cause‐specific hospitalisation including sepsis	Primary sepsis; possible Australian overlap; moderate
Hu et al. (2024) [24]	Taiwan; NHIRD	Propensity‐score matched; non‐SGLT2i comparator	Sepsis/septic shock	Narrative; serious
Henney et al. (2025) [25]	Multinational; TriNetX EHR network	Active‐comparator new‐user (index date 2 years after initiation), propensity‐score matched; DPP‐4i	Incident pneumonia; severe sepsis	Primary pneumonia and sepsis; moderate
Maki et al. (2025) [26]	Japan; DPC inpatient database	Hospitalised‐pneumonia cohort, GEE; DPP‐4i	In‐hospital outcomes after pneumonia	Narrative prognosis; moderate
Ulambayar et al. (2025) [27]	Hungary; Debrecen registry	Longitudinal cohort; multiple drug classes	Sepsis	Narrative; serious
Wu et al. (2026) [28]	Taiwan; NHIRD	Active‐comparator new‐user, 1:2 matched; DPP‐4i	Sepsis; septic shock; all‐cause mortality	Primary sepsis; all‐cause mortality narrative; moderate
Alfonso Arvez et al. (2026) [29]	Australia; VAED‐PBS‐NDI	Active‐comparator post‐discharge, IPTW; DPP‐4i	Overall and infection‐specific hospitalisation	Primary pneumonia and sepsis; hospitalisation (not pooled); possible Australian overlap; moderate
Jhu et al. (2026) [30]	United States; TriNetX EHR network (ESKD)	Target‐trial emulation, active‐comparator new‐user, propensity‐score matched; DPP‐4i	Sepsis 0.87; pneumonia 0.95	Overlap‐inclusive sensitivity; moderate
Chen et al. (2026) [31]	Global; TriNetX EHR network (peritoneal dialysis)	Target‐trial emulation, propensity‐score matched; non‐user comparator	Pneumonia 0.66; sepsis 0.66	Narrative; serious
Cheng et al. (2026) [32]	United States; TriNetX EHR network (psoriasis)	Target‐trial emulation, active‐comparator new‐user, propensity‐score matched; DPP‐4i	Severe sepsis 0.69 vs. DPP‐4i; 1.03 vs. GLP‐1 RA	Overlap‐inclusive sensitivity (sepsis); comparator contrast; moderate
Hsiao et al. (2026) [33]	United States; TriNetX EHR network (advanced chronic kidney disease)	Active‐comparator new‐user, propensity‐score matched; GLP‐1 RA comparator	Pneumonia 0.98; sepsis, no difference	Narrative (non‐DPP‐4i comparator); moderate

Abbreviations: CDARS, Clinical Data Analysis and Reporting System; CPRD, Clinical Practice Research Datalink; DPC, Diagnosis Procedure Combination; DPP‐4i, dipeptidyl peptidase‐4 inhibitor; EHR, electronic health record; ESKD, end‐stage kidney disease; GEE, generalised estimating equations; GLP‐1 RA, glucagon‐like peptide‐1 receptor agonist; HES, Hospital Episode Statistics; ICU, intensive care unit; IPTW, inverse probability of treatment weighting; NDI, National Death Index; NDSS, National Diabetes Services Scheme; NHIRD, National Health Insurance Research Database; PBS, Pharmaceutical Benefits Scheme; ROBINS‐I, Risk Of Bias In Non‐randomised Studies of Interventions; SGLT2i, sodium‐glucose cotransporter 2 inhibitor; VAED, Victorian Admitted Episodes Dataset.

All 11 studies contributing to quantitative synthesis were judged to have moderate risk of bias, and no study was judged to have low risk (Sections S4, S5 and Tables S5, S6). The principal concerns were residual confounding by frailty, kidney function, diabetes severity, prior infection, vaccination, cardiopulmonary reserve and healthcare access. Certainty was very low for incident pneumonia and sepsis and low for infection‐related hospitalisation (Section S8 and Table S14).

### 3.2 Co‐Primary Outcomes and Infection‐Related Hospitalisation

Five DPP‐4i active‐comparator studies contributed to the incident‐pneumonia synthesis [13, 18, 21, 25, 29]. The pooled estimate was lower among SGLT2i users (0.65, 95% CI: 0.55–0.75), with substantial heterogeneity (*I* ^2^ = 89.8%; τ^2^ = 0.010, 95% CI: 0.002–0.170) and a 95% prediction interval of 0.45–0.93 (Table 2; Figure 2A); certainty was very low. Five studies contributed to incident sepsis [13, 23, 25, 28, 29]. For incident sepsis, the 95% prediction interval was 0.38–1.13 and crossed 1.0; the exploratory pooled mean was 0.65 (95% CI: 0.53–0.80; *I* ^2^ = 90.6%; τ^2^ = 0.024, 95% CI: 0.007–0.225; very low certainty) (Figure 2B). Because heterogeneity persisted after every omission and no single study was influential (Table S12), this exploratory pooled mean describes an average across heterogeneous settings rather than a common effect.

**TABLE 2:** Pooled associations between SGLT2 inhibitor use and severe infection outcomes versus DPP‐4 inhibitors.

Outcome	Studies, *n*	REML‐modified Hartung‐Knapp estimate (95% CI)	95% prediction interval	*I* ^2^, % (τ^2^)
Incident pneumonia (co‐primary; very low certainty)	5	0.65 (0.55–0.75)	0.45–0.93	89.8 (0.010)
Incident pneumonia, leave‐one‐out omitting the federated‐network cohort	4	0.61 (0.56–0.67)	0.54–0.69	0.0 (< 0.001)
Incident sepsis (co‐primary; exploratory pooled synthesis; very low certainty)	5	0.65 (0.53–0.80)	0.38–1.13	90.6 (0.024)
Infection‐related hospitalisation (secondary; low certainty)^a^	2	Not pooled^a^	—	—
Incident pneumonia, overlap‐inclusive sensitivity	6	0.68 (0.55–0.85)	0.39–1.21	93.1 (0.035)
Incident sepsis, overlap‐inclusive sensitivity	7	0.68 (0.58–0.81)	0.43–1.08	89.4 (0.027)

*Note:* Estimates were pooled on the natural‐log scale and restricted to DPP‐4i active‐comparator studies. Overlap‐inclusive analyses additionally included the federated‐network cohorts restricted to end‐stage kidney disease (both endpoints) and psoriasis (sepsis only). The prespecified pneumonia leave‐one‐out analysis is displayed because omission of the multinational federated‐network cohort eliminated the observed heterogeneity. For sepsis, *I* ^2^ remained between 88.1% and 92.4% across leave‐one‐out analyses; full results are provided in Table S8. Mortality and ICU evidence were not pooled and are summarised narratively in Results 3.4. Pooled estimates combine hazard ratios and incidence rate ratios as a pragmatic synthesis of related but non‐identical estimands; effect‐measure‐stratified results are in Table S11. *I* ^2^ was calculated from Cochran's Q. Certainty of evidence was rated with GRADE (Table S14).

Abbreviations: CI, confidence interval; REML, restricted maximum likelihood.

^a^ Two studies (Narasaki 2024, HR 0.74, 95% CI: 0.67–0.81; Alfonso Arvez 2026, weighted IRR 0.74, 0.71–0.77) were not pooled because fewer than three studies were available (registered protocol); event structures also differed (Table S10); see Results 3.2 and Table S9.

**FIGURE 2:**
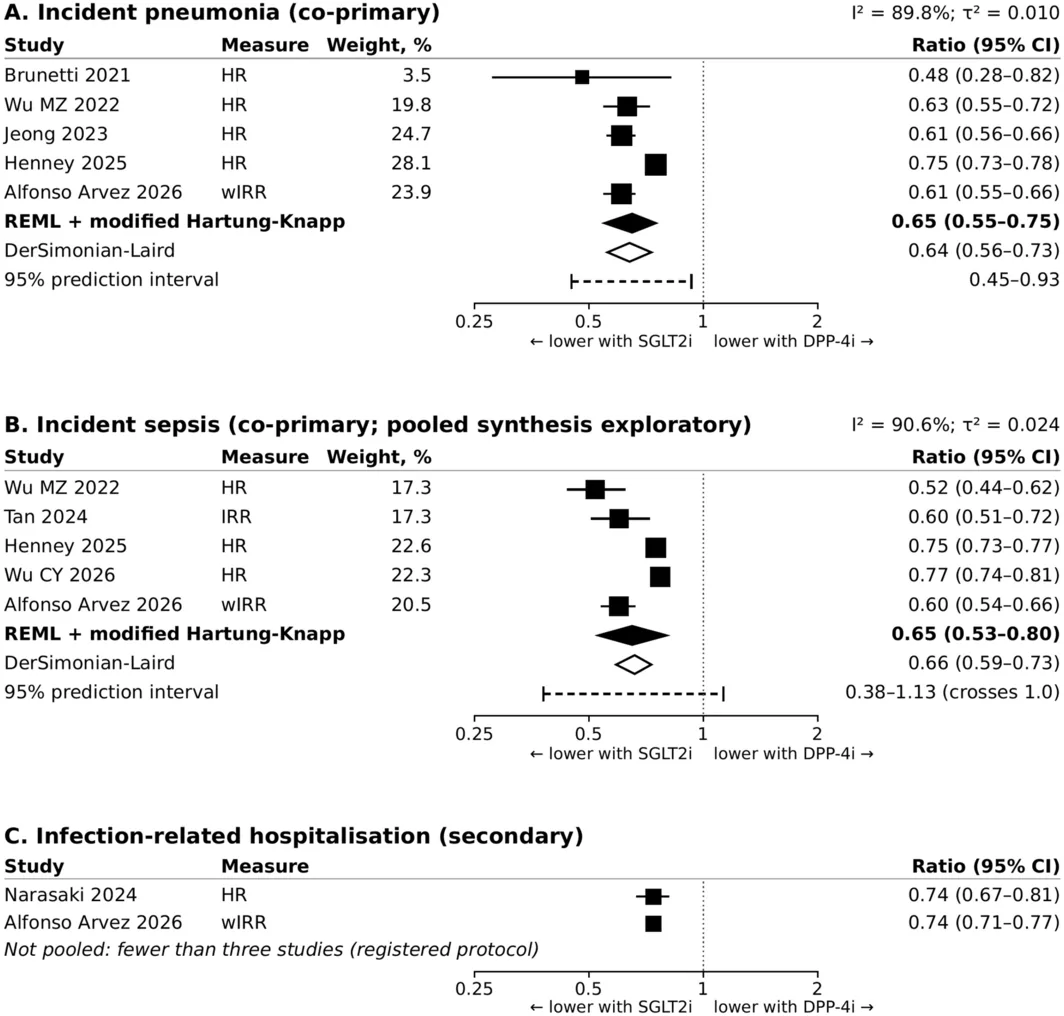
Forest plots for SGLT2 inhibitors versus DPP‐4 inhibitors. (A) Incident pneumonia; (B) incident sepsis (prespecified co‐primary outcome; pooled synthesis interpreted as exploratory); (C) infection‐related hospitalisation. Panels A and B show REML estimates with modified Hartung‐Knapp confidence intervals, DerSimonian‐Laird estimates and 95% prediction intervals; panel C shows the two contributing studies, which were not pooled because fewer than three studies were available. The effect measure reported by each study and, in panels A and B, its random‐effects weight are shown. Squares represent study‐specific adjusted ratio estimates (size proportional to weight in panels A and B; uniform in panel C); horizontal lines are 95% confidence intervals; filled diamonds are primary pooled estimates; open diamonds are DerSimonian‐Laird estimates; and dashed bars are prediction intervals. *I* ^2^ was calculated from Cochran's Q. Ratio estimates below 1.0 indicate lower estimates among SGLT2 inhibitor users. CI, confidence interval; DPP‐4i, dipeptidyl peptidase‐4 inhibitor; HR, hazard ratio; IRR, incidence rate ratio; REML, restricted maximum likelihood; SGLT2i, sodium‐glucose cotransporter 2 inhibitor; wIRR, weighted incidence rate ratio.

Two studies reported infection‐related hospitalisation: Narasaki 2024 (HR 0.74, 95% CI: 0.67–0.81; time to first event) and Alfonso Arvez 2026 (weighted IRR 0.74, 0.71–0.77; event rate including repeat admissions) [22, 29]. In accordance with the registered protocol for outcomes with fewer than three studies, these estimates were not pooled (Figure 2C); certainty was low. Tan 2024 did not report overall infection‐related hospitalisation; its closest category, ICD‐10‐AM chapter A as primary diagnosis (IRR 0.68, 0.63–0.74), excludes respiratory, urinary and skin infections and is reported only as contextual evidence, not quantitatively combined with the two eligible overall infection‐related hospitalisation estimates (Table S9) [23]. These estimates should be interpreted as associations because hospitalisation reflects admission thresholds and coding practice in addition to infection occurrence and severity.

### 3.3 Sensitivity Analyses and Overlapping Cohorts

Prespecified leave‐one‐out analyses (Section S6) showed different heterogeneity patterns across the co‐primary outcomes. For incident pneumonia, omitting the multinational federated‐network cohort reduced *I* ^2^ from 89.8% to 0.0% and yielded 0.61 (95% CI: 0.56–0.67; prediction interval 0.54–0.69); omitting any other cohort left *I* ^2^ between 87.6% and 92.0%. For incident sepsis, *I* ^2^ remained between 88.1% and 92.4% after each omission. Thus, pneumonia heterogeneity was concentrated in one cohort, whereas sepsis heterogeneity was not explained by any single study (Table 2; Tables S7, S8 and S12). The high pneumonia *I* ^2^ should be interpreted alongside the modest τ^2^ (0.010; corresponding to a between‐study standard deviation of approximately 0.10 on the log scale): the exceptionally small within‐study variance of the multinational federated‐network estimate made *I* ^2^ highly sensitive to modest differences among estimates that all remained below 1.0. By contrast, sepsis showed a larger τ^2^ (0.024) and a prediction interval crossing 1.0, indicating greater dispersion in the underlying associations rather than precision alone. Substituting the alternative Hong Kong cohort for the retained CDARS study gave 0.66 (0.57–0.77; *I* ^2^ = 88.9%) [19]. For sepsis, substituting the ICU‐admission‐for‐sepsis estimate (HR 0.61, 95% CI: 0.43–0.85) from the overlapping CDARS cohort for Wu MZ 2022 gave 0.68 (0.57–0.81; *I* ^2^ = 85.5%; prediction interval 0.44–1.05) [14]. Adding the ESKD TriNetX cohort to pneumonia changed the estimate from 0.65 to 0.68 (0.55–0.85) and moved the prediction interval across the null (0.39–1.21) [30]. Adding the ESKD and psoriasis cohorts to sepsis yielded 0.68 (0.58–0.81; *I* ^2^ = 89.4%; τ^2^ = 0.027), with a prediction interval of 0.43–1.08 [30, 32]. Similar pooled means can therefore coexist with materially different predictive uncertainty.

Restricting analyses to hazard ratios gave 0.65 (95% CI: 0.53–0.81; *I* ^2^ = 89.0%) for pneumonia and 0.68 (0.40–1.15; *I* ^2^ = 89.4%) for sepsis; the sepsis estimate was imprecise with three studies. Incidence‐rate‐ratio strata contained one or two studies (Table S11). No difference between effect‐measure strata was detected for pneumonia (*p* = 0.40) or sepsis (*p* = 0.33), although these tests had little power. Tan 2024's ICD‐10‐AM chapter A estimate was retained as contextual evidence only and was not quantitatively combined with the two overall infection‐related hospitalisation estimates because its outcome definition was not equivalent (Table S9).

An exploratory post hoc grouping by data‐source architecture was confounded by population, outcome definition and study design, and no interaction test was performed; it is reported in Section S11, Section S12 and Tables S16, S17 as hypothesis‐generating only and is not used to explain heterogeneity. The multinational federated‐network pneumonia cohort also differed from the four single‐jurisdiction cohorts in design, most notably an index date 2 years after drug initiation (Section S14 and Table S18).

### 3.4 Mortality, Critical Illness and Non‐Pooled Evidence

Mortality evidence was heterogeneous in endpoint definition and was therefore not pooled. Wu MZ 2022 reported lower infection‐related mortality (HR 0.43, 95% CI: 0.32–0.57), pneumonia‐related mortality (HR 0.41, 95% CI: 0.29–0.58) and sepsis‐related mortality (HR 0.39, 95% CI: 0.18–0.84) among SGLT2i users [13]. Wu CY 2026 reported lower all‐cause mortality (HR 0.60, 95% CI: 0.54–0.66), which was not eligible as an infection‐specific endpoint [28]. In a cohort of older adults already hospitalised with pneumonia, Maki 2025 found no statistically significant difference in post‐pneumonia in‐hospital mortality (OR 0.64, 95% CI: 0.40–1.05) [26]. These estimates addressed distinct outcomes and time origins and were interpreted narratively. One Hong Kong cohort reported lower ICU admission with SGLT2i (0.79, 0.69–0.91) [14]. Studies using non‐active or mixed comparators remained narrative [20, 24, 27, 31, 33]. In a TriNetX study restricted to psoriasis with T2DM, severe‐sepsis estimates were lower with SGLT2i than with DPP‐4i (0.69, 0.59–0.80) but not in a separately matched comparison with GLP‐1 receptor agonists (1.03, 0.89–1.18) [32]. In another TriNetX cohort of 22,393 matched pairs with advanced chronic kidney disease, SGLT2i and GLP‐1 receptor agonists did not differ for pneumonia (0.98, 0.92–1.04) or sepsis [33]. These selected‐population comparisons were used for triangulation rather than direct generalisation to the broader T2DM population. One Australian cohort reported higher hospitalisation for genital mycotic infection, foot infection and osteomyelitis despite lower estimates for several severe infection endpoints [29].

## 4 Discussion

This review supports a narrow conclusion: the available evidence did not suggest an increased average risk of severe infection with SGLT2i relative to DPP‐4i, but the lower pooled estimates should not be interpreted as preventive effects. Pneumonia heterogeneity was concentrated in one multinational federated‐network cohort, whereas sepsis remained highly heterogeneous after every study omission and its prediction interval crossed the null. Accordingly, although incident sepsis remained a prespecified co‐primary outcome, its pooled quantitative synthesis is interpreted as exploratory. Cross‐design triangulation raised, but did not establish, comparator dependence and residual treatment channelling; much of that evidence came from clinically selected populations that were not directly transportable to the broader T2DM population.

The greater instability of sepsis estimates is clinically and methodologically plausible. Sepsis is a syndrome rather than an anatomical diagnosis, and its ascertainment depends on infection source, evolving definitions, microbiological testing, coding practice, organ‐dysfunction thresholds and intensive‐care triage. Pneumonia is also susceptible to misclassification, but hospital and claims algorithms may capture a more consistent phenotype. Henney 2025 was not only the most precise study; its point estimate (0.75) also lay above the 0.48–0.63 range of the other four pneumonia cohorts, so the fall in *I* ^2^ after omission reflects estimate‐level separation as well as statistical weight. It also differed in design, most notably an index date 2 years after drug initiation, and in setting and outcome ascertainment (Table S18); which of these differences accounts for the separation cannot be determined from five studies. Given this difference in time zero, the primary pneumonia estimate (0.65) should be read together with the single‐jurisdiction estimate (0.61, *I* ^2^ = 0%). The defensible conclusion is therefore endpoint‐specific: pneumonia heterogeneity in this dataset was concentrated in one cohort, whereas sepsis heterogeneity was not explained by any single study.

This review is best understood as an updated and methodologically refined synthesis rather than as an answer to a new question. The previous review already reported lower observational estimates for pneumonia, pneumonia‐related mortality and sepsis [10]. The present review adds cohorts published after that review's search, includes five DPP‐4i active‐comparator cohorts for each co‐primary endpoint, restricts all principal pooling to DPP‐4i comparisons, separates incident infection from post‐infection prognosis, makes data‐source overlap explicit, and reports prediction intervals and GRADE ratings. Restriction to DPP‐4i does not eliminate confounding, but it better approximates a routine clinical choice between two oral drug classes than non‐user comparisons.

Prediction intervals qualify pooled confidence intervals. A confidence interval describes uncertainty around the mean association, whereas a prediction interval estimates the dispersion expected in a future study exchangeable with those included under the fitted random‐effects model [17]. The primary pneumonia interval remained below 1.0, the sepsis interval crossed 1.0, and overlap‐inclusive analyses moved both across the null. With only five to seven cohorts, these limits are themselves imprecise, but they show why narrow pooled confidence intervals should not be equated with uniform effects across settings.

Comparator choice provides an additional, indirect interpretive check. In two general T2DM cohorts that also compared SGLT2i with GLP‐1 receptor agonists, comparisons did not show a consistent or statistically clear lower‐risk pattern with SGLT2i (hospitalised community‐acquired pneumonia HR 0.94, 95% CI: 0.44–1.89 [18]; incident pneumonia HR 1.27, 1.23–1.32, and severe sepsis HR 1.27, 1.22–1.31 [25]). In two clinically selected populations (psoriasis and advanced chronic kidney disease), SGLT2i and GLP‐1 receptor agonists also did not differ [32, 33]. Because GLP‐1 receptor agonists may themselves be associated with lower infection risk, these contrasts cannot separate residual channelling against DPP‐4i from a specific association with GLP‐1 receptor agonists, and they provide qualitative triangulation rather than transportable estimates. Randomised evidence has not demonstrated a consistent class‐wide preventive effect. A meta‐analysis of trials directly comparing SGLT2i with DPP‐4i did not report pneumonia or sepsis; its estimate for upper respiratory tract infection was below unity but not statistically significant (RR 0.78, 95% CI: 0.61–1.02) [34]. A network meta‐analysis of 105 trials found no consistent class‐level reduction in infection outcomes, although high‐dose canagliflozin was associated with a lower risk of sepsis in six trials (OR 0.57, 95% CI: 0.35–0.91) [35]. Together, these observations raise the possibility that treatment channelling and residual confounding by frailty, physiological reserve and healthcare engagement contribute to the lower observational estimates. They do not exclude a pharmacological contribution, but they argue against interpreting the pooled estimates as anti‐infective effects.

A pharmacological contribution remains possible because SGLT2i reduce heart‐failure events and slow kidney disease progression, potentially preserving physiological reserve during infection [5, 6]. However, propensity‐score methods cannot balance unmeasured functional status, time‐varying glycaemia, vaccination, recent infection burden or frailty, and treatment groups may differ in healthcare engagement.

The findings also argue against treating infection as a single class‐wide safety outcome. The Australian post‐discharge cohort observed lower hospitalisation for pneumonia and sepsis but higher hospitalisation for genital mycotic infection, foot infection and osteomyelitis [29]. Communication about SGLT2i safety should distinguish anatomical site and severity rather than implying that one signal applies to all infections.

For practice, the defensible interpretation is narrow. The evidence does not justify prescribing SGLT2i to prevent pneumonia or sepsis and does not modify established cardiorenal indications, contraindications or sick‐day guidance. It does indicate that choosing SGLT2i rather than DPP‐4i was not associated with an increased average risk of severe infection in the populations studied. Established risks such as genital mycotic infection and ketoacidosis remain actively managed.

Strengths include PROSPERO registration before a final four‐database search through 29 June 2026, restriction of principal pooling to a clinically relevant active comparator, endpoint‐specific mapping, explicit handling of overlapping data sources, prespecified leave‐one‐out analyses, effect‐measure‐stratified analyses, REML with modified Hartung‐Knapp inference and prediction intervals, ROBINS‐I, GRADE, and triangulation against alternative‐comparator and randomised evidence.

Limitations are substantial. All evidence was observational; no endpoint was supported by many independent cohorts; outcome definitions, follow‐up and ratio measures differed; and frailty, vaccination, adherence, socioeconomic position, time‐varying glycaemia, kidney function, and prior infection burden were incompletely captured. Hazard ratios and incidence rate ratios are related but non‐identical estimands. The two infection‐related hospitalisation estimates, which also differed in event structure, were reported without pooling because fewer than three studies remained. Patient‐level overlap could not be quantified for Australian or federated‐network studies. Prediction intervals based on few studies are imprecise and assume an exchangeable random‐effects distribution. Mortality evidence was sparse and clinically heterogeneous and therefore was not pooled; Egger testing was underpowered. Database indexing lag may still have affected retrieval. Finally, the data‐source assessment was post hoc and could not separate architecture from case‐mix, outcome definition or study design.

Future comparative‐effectiveness studies should emulate target trials with prespecified eligibility, index dates, censoring rules and negative‐control outcomes, should report validated severe‐infection algorithms and data‐source provenance, and should include more than one active comparator, since a single comparator cannot distinguish drug effect from channelling. Pragmatic or registry‐based trials with prespecified severe‐infection outcomes would be required before any preventive claim.

In conclusion, observational active‐comparator evidence did not suggest an increased average risk of severe infection with SGLT2i compared with DPP‐4i. Certainty was very low for incident pneumonia and sepsis and low for infection‐related hospitalisation; for sepsis, the pooled synthesis is exploratory in interpretation. The lower pooled estimates were heterogeneous and sensitive to comparator choice, and they should not be interpreted as a preventive effect.

## Author Contributions

**Yung‐Ming Huang:** conceptualization, data curation, writing – original draft, writing – review and editing. **Jen‐Hung Wang:** formal analysis, methodology, writing – review and editing. **Wan‐Ting Huang:** data curation, methodology, writing – review and editing. **Ji‐Ze Hsu:** data curation, methodology, writing – review and editing. **Soon‐Hian Teh:** data curation, conceptualization, methodology, project administration, supervision, validation, writing – original draft, writing – review and editing.

## Ethics Statement

This systematic review and meta‐analysis synthesised published aggregate data and involved no identifiable individual‐level data. Institutional review board approval and informed consent were therefore not required.

## Conflicts of Interest

The authors declare no conflicts of interest.

## Supporting information


**Figure S1:** Exploratory funnel plots.
**Table S1:** Database search strategies.
**Table S2:** Full‐text articles excluded with reasons, grouped according to the Figure 1 exclusion categories.
**Table S3:** Endpoint mapping and overlap‐handling rules.
**Table S4:** Data‐source overlap matrix and endpoint‐prioritisation rationale.
**Table S5:** ROBINS‐I domain‐level risk of bias.
**Table S6:** ROBINS‐I overall risk of bias and rationale.
**Table S7:** Incident pneumonia sensitivity analyses.
**Table S8:** Incident sepsis sensitivity analyses (prespecified co‐primary outcome; pooled synthesis interpreted as exploratory).
**Table S9:** Infection‐related hospitalisation: contributing estimates and contextual evidence.
**Table S10:** Effect measure, model and event structure of studies contributing to the principal analyses.
**Table S11:** Analyses stratified by effect measure (REML, modified Hartung‐Knapp).
**Table S12:** Study weights, influence diagnostics and complete leave‐one‐out results (REML, modified Hartung‐Knapp).
**Table S13:** REML‐modified Hartung‐Knapp estimates and 95% prediction intervals.
**Table S14:** GRADE certainty of evidence.
**Table S15:** Exploratory Egger regression tests for small‐study effects.
**Table S16:** Structured heterogeneity and clinical characterisation mapping for primary endpoints.
**Table S17:** Exploratory post hoc stratification of the co‐primary endpoints by data‐source architecture.
**Table S18:** Design of the multinational federated‐network cohort compared with the four single‐jurisdiction pneumonia cohorts.

## Data Availability

All data analysed were extracted from published studies cited in the References and are summarised in the main manuscript and Appendix S1. The extracted dataset and analysis code are available from the corresponding author on reasonable request.
