# Supplementary material for: Severe Infection Outcomes With SGLT2 Versus DPP‐4 Inhibitors in Type 2 Diabetes: A Systematic Review and Active‐Comparator Meta‐Analysis of Real‐World Cohorts
Source: Endocrinol Diabetes Metab. 2026 Nov 1;9(6):e70381. doi: 10.1002/edm2.70381 (PMC13724236; doi:10.1002/edm2.70381)
Supplement: Supplementary file 1 [file EDM2-9-e70381-s001.docx]

**Supplementary Appendix**

Severe Infection Outcomes With SGLT2 Versus DPP-4 Inhibitors in Type 2 Diabetes: A Systematic Review and Active-Comparator Meta-Analysis of Real-World Cohorts

Yung-Ming Huang, MS, RPh; Jen-Hung Wang, PhD; Wan-Ting Huang, MS; Ji-Ze Hsu, MS; Soon-Hian Teh, MD

Prepared for submission to Endocrinology, Diabetes & Metabolism. Review registration: PROSPERO CRD420261390509.

# Contents

This appendix contains database search strategies (Table S1), full-text exclusions with reasons (Table S2), endpoint mapping and overlap-handling rules (Table S3), a data-source overlap matrix (Table S4), ROBINS-I assessments (Tables S5-S6), sensitivity analyses (Tables S7-S9), effect measure and event structure of contributing studies (Table S10), effect-measure-stratified analyses (Table S11), study weights and influence diagnostics (Table S12), REML-modified Hartung-Knapp estimates and prediction intervals (Table S13), a GRADE profile (Table S14), reporting-guideline information (Section S9), exploratory publication-bias assessment (Table S15 and Supplementary Fig. S1), structured heterogeneity mapping (Table S16), a post hoc data-source assessment (Table S17), recently identified cohorts and their analytic role (Section S13), a design comparison of the multinational federated-network pneumonia cohort with the single-jurisdiction cohorts (Section S14, Table S18), and abbreviations.

# Section S1. Database Search Strategies

The final PubMed, Embase, Scopus and Cochrane CENTRAL searches were completed on 29 June 2026, without language or publication-date restrictions. Individual agent names were included to capture reports predating indexing under class terms. The searches retrieved 479 records: PubMed 171, Embase 136, Scopus 153 and CENTRAL 19. After 77 duplicates were removed, 402 records were screened. Complete search strings and platform details are shown below.

**Table S1. Database search strategies.**

| **Database / source** | **Platform / method** | **Search date** | **Search string / procedure description** | **Records retrieved** |
| --- | --- | --- | --- | --- |
| PubMed | PubMed (NLM) | 29 June 2026 | (("Sodium-Glucose Transporter 2 Inhibitors"[Mesh] OR "SGLT2 inhibitor*"[tiab] OR "SGLT-2 inhibitor*"[tiab] OR "sodium glucose cotransporter 2 inhibitor*"[tiab] OR "sodium-glucose cotransporter-2 inhibitor*"[tiab] OR dapagliflozin[tiab] OR empagliflozin[tiab] OR canagliflozin[tiab] OR ertugliflozin[tiab] OR sotagliflozin[tiab] OR ipragliflozin[tiab] OR tofogliflozin[tiab] OR luseogliflozin[tiab]) AND ("Diabetes Mellitus, Type 2"[Mesh] OR "type 2 diabetes"[tiab] OR "T2D"[tiab] OR "T2DM"[tiab]) AND (infection*[tiab] OR pneumonia[tiab] OR sepsis[tiab] OR "septic shock"[tiab] OR hospitali*[tiab] OR ICU[tiab] OR "intensive care"[tiab] OR mortality[tiab] OR death[tiab])) NOT (animals[mh] NOT humans[mh]) | 171 |
| Embase | Elsevier (Embase.com) | 29 June 2026 | ('sodium glucose cotransporter 2 inhibitor'/exp OR 'sglt2 inhibitor*':ti,ab OR 'sglt-2 inhibitor*':ti,ab OR dapagliflozin:ti,ab OR empagliflozin:ti,ab OR canagliflozin:ti,ab OR ertugliflozin:ti,ab OR sotagliflozin:ti,ab OR ipragliflozin:ti,ab OR tofogliflozin:ti,ab OR luseogliflozin:ti,ab) AND ('type 2 diabetes mellitus'/exp OR 'type 2 diabetes':ti,ab OR t2d:ti,ab OR t2dm:ti,ab) AND ('infection'/exp OR infection*:ti,ab OR pneumonia:ti,ab OR sepsis:ti,ab OR 'septic shock':ti,ab OR hospitali*:ti,ab OR 'intensive care':ti,ab OR icu:ti,ab OR mortality:ti,ab OR death:ti,ab) AND [humans]/lim | 136 |
| Scopus | Scopus (Elsevier) | 29 June 2026 | TITLE-ABS-KEY(("SGLT2 inhibitor*" OR "SGLT-2 inhibitor*" OR "sodium glucose cotransporter 2 inhibitor*" OR "sodium-glucose cotransporter-2 inhibitor*" OR dapagliflozin OR empagliflozin OR canagliflozin OR ertugliflozin OR sotagliflozin OR ipragliflozin OR tofogliflozin OR luseogliflozin) AND ("type 2 diabetes" OR T2D OR T2DM) AND (infection* OR pneumonia OR sepsis OR "septic shock" OR hospitali* OR "intensive care" OR ICU OR mortality OR death)) | 153 |
| Cochrane CENTRAL | Cochrane Library (Wiley) | 29 June 2026 | ((SGLT2 OR SGLT-2 OR "sodium glucose cotransporter 2" OR "sodium-glucose cotransporter-2" OR dapagliflozin OR empagliflozin OR canagliflozin OR ertugliflozin OR sotagliflozin OR ipragliflozin OR tofogliflozin OR luseogliflozin) AND ("type 2 diabetes" OR T2D OR T2DM) AND (infection OR pneumonia OR sepsis OR "septic shock" OR hospitalization OR hospitalisation OR "intensive care" OR ICU OR mortality OR death)) | 19 |
| Total |  |  |  | 479 |

# Section S2. Full-Text Articles Excluded with Reasons

Thirty-nine full-text reports were assessed for eligibility; 21 were excluded and 18 studies were included in the qualitative synthesis. Eleven studies contributed estimates to quantitative analyses, 10 of them to at least one pooled analysis. Each excluded report was assigned one mutually exclusive primary exclusion category corresponding to Figure 1.

Table S2. Full-text articles excluded with reasons, grouped according to the Fig. 1 exclusion categories.

| **No.** | **First author/year** | **Country** | **Publication type** | **Exclusion reason** | **Justification** |
| --- | --- | --- | --- | --- | --- |
| 1 | Narasaki 2022 | United States | Conference abstract | Conference abstract or superseded abstract | Abstract superseded by Narasaki 2024 full article (Lancet Regional Health - Americas). Retain full publication only. |
| 2 | Rim 2021 | United States | Conference abstract | Conference abstract or superseded abstract | Abstract superseded by Rim 2022 full article (American Journal of the Medical Sciences). |
| 3 | Lim 2024 | Korea | Full article | COVID-specific cohort or wrong clinical context | Primary outcome was MACCE in COVID-19 patients; pneumonia endpoint was COVID-19-specific, not general incident pneumonia/sepsis in outpatient T2DM. |
| 4 | Hsin-Chieh 2022 | Taiwan | Full article | COVID-specific cohort or wrong clinical context | COVID-19 mortality/ICU/intubation prognosis; not incident severe infection in general T2DM outpatients. |
| 5 | Khunti 2022 | United Kingdom | Full article | COVID-specific cohort or wrong clinical context | Hospitalized COVID-19 mortality study; not general pneumonia/sepsis/infection-hospitalization endpoint. |
| 6 | Mannucci 2022 | Italy | Full article | COVID-specific cohort or wrong clinical context | COVID-19 surveillance mortality; mixed comparator not suitable for DPP-4i active-comparator infection synthesis. |
| 7 | Monda 2023 | Italy | Full article | COVID-specific cohort or wrong clinical context | In-hospital death in hospitalized COVID-19; not incident pneumonia or sepsis. |
| 8 | Ouchi 2022 | Japan | Full article | COVID-specific cohort or wrong clinical context | Severe COVID-19 outcome; not routine non-COVID severe infection endpoint. |
| 9 | Ferrannini 2022 | Italy | Full article | COVID-specific cohort or wrong clinical context | Incident severe COVID-19; not compatible with primary endpoint mapping. |
| 10 | Sainsbury 2021 | Australia | Full article | COVID-specific cohort or wrong clinical context | COVID-19 diagnosis/prognosis; not pneumonia, sepsis, or infection-related hospitalization in general T2DM population. |
| 11 | Dave 2019 | United States | Full article | Wrong outcome for current review question | Outcome was severe UTI/pyelonephritis/sepsis with UTI; outside the review's severe infection focus. |
| 12 | Akkus 2024 | Turkey | Full article | No suitable active comparator or mixed non-comparative design | Prospective cohort focused on pyuria/bacteriuria and UTI risk, without an eligible severe infection endpoint or DPP-4i active-comparator contrast for this review. |
| 13 | Alhefel 2026 | Qatar | Full article | No suitable active comparator or mixed non-comparative design | UTI prevalence and predictors among SGLT2i users; not a severe infection endpoint and no suitable active-comparator contrast for pooling. |
| 14 | UTI continuation 2025 | Mixed | Full article | Wrong outcome for current review question | Primary outcomes were cardiovascular/renal outcomes after UTI and recurrent UTI risk; not prespecified severe infection endpoint. |
| 15 | Hertel 2025 | Germany | Full article | Wrong outcome for current review question | Fournier gangrene safety endpoint; localized genital/perineal infection rather than a prespecified severe infection endpoint. |
| 16 | Dull 2019 | United States | Case report | No suitable active comparator or mixed non-comparative design | Two-case report of euglycemic DKA precipitated by infection; no cohort comparator or denominator. |
| 17 | Alfonso Arvez 2025 | Australia | Systematic review | Review-level evidence, ineligible as primary study | Secondary evidence from a prior systematic review and meta-analysis; original cohort reports, rather than review-level data, were retained or assessed elsewhere. |
| 18 | Systematic review 2021 | Mixed | Systematic review | Review-level evidence, ineligible as primary study | Secondary evidence from a systematic review; review-level data were not treated as a unique original cohort report eligible for pooling. |
| 19 | Pediatric HF study | Mixed | Full article | Other prespecified reason | Pediatric heart failure population; not adults with T2DM and not a severe infection outcome eligible for this review. |
| 20 | Zeng 2026 | Mixed | Systematic review and meta-analysis | Review-level evidence, ineligible as primary study | Network meta-analysis of randomised trials; retained for external interpretation but ineligible as a primary real-world cohort. |
| 21 | Li 2026 | Mixed | Systematic review and meta-analysis | Review-level evidence, ineligible as primary study | Meta-analysis of randomised controlled trials; retained for external interpretation but ineligible as a primary real-world cohort. |

# Section S3. Endpoint Mapping and Overlap Handling

The table below maps each included study to the endpoints it contributed and specifies its role in the synthesis. Where multiple studies used substantially overlapping data sources, the more endpoint-specific, active-comparator, incident-user estimate was prioritized. Alternative estimates were retained for sensitivity or narrative interpretation. All required aggregate data were available from published reports and supplementary materials; study authors were therefore not contacted.

**Table S3. Endpoint mapping and overlap-handling rules.**

| **Study** | **Country / data source** | **Incident pneumonia** | **Incident sepsis** | **Infection hospitalization** | **Mortality** | **Overlap / handling note** |
| --- | --- | --- | --- | --- | --- | --- |
| Brunetti 2021 | United Kingdom / CPRD-HES | Primary | - | - | - | No overlap identified. |
| Wu MZ 2022 | Hong Kong / CDARS | Primary | Primary | - | Infection-related mortality (narrative) | CDARS overlap with Au 2022 and Ng 2023. Wu MZ 2022 prioritized for pneumonia and sepsis primary pools; Au 2022 retained as sensitivity only. |
| Au 2022 | Hong Kong / CDARS | Sensitivity only | - | - | Pneumonia mortality (narrative) | Excluded from pneumonia primary pool due to CDARS overlap with Wu MZ 2022. |
| Jeong 2023 | South Korea / HIRA | Primary | - | - | - | No overlap identified. |
| Ng 2023 | Hong Kong / CDARS | - | - | - | ICU and all-cause mortality (narrative) | CDARS-related; ICU admission not poolable; retained for narrative critical-illness summary. ICU admission for sepsis used only in a substitution sensitivity analysis for Wu MZ 2022 (Table S8). |
| Henney 2025 | Multinational / TriNetX EHR | Primary | Primary | - | - | Main statistical contributor to pneumonia heterogeneity; I²=0% when omitted. Retained in the primary pool because it represents the broadest active-comparator T2DM cohort in this network. |
| Narasaki 2024 | United States / Veterans Affairs | - | - | Not pooled (k=2) | - | No overlap identified. |
| Tan 2024 | Australia / NDSS-PBS-NDI | - | Primary | Contextual only (not eligible for overall endpoint pooling) | - | Possible partial overlap with Alfonso Arvez 2026 (both Australian PBS). Overlap-removal sensitivity conducted for eligible endpoints. Tan 2024 did not report overall infection-related hospitalisation; its ICD-10-AM chapter A estimate (primary diagnosis) is reported descriptively only and was not quantitatively combined with the overall infection-related hospitalisation estimates. |
| Hu 2024 | Taiwan / NHIRD | - | Narrative (non-active comparator) | - | - | Non-active comparator; narrative only. |
| Maki 2025 | Japan / DPC | - | - | - | Pneumonia-related mortality (narrative) | Inpatient pneumonia prognosis design; not pooled with incident pneumonia. |
| Ulambayar 2025 | Hungary / Registry | - | Narrative (non-active comparator) | - | - | Non-active comparator; narrative only. |
| Wu CY 2026 | Taiwan / NHIRD | - | Primary | - | All-cause mortality (narrative) | Different NHIRD time window from Hu 2024; not considered overlapping for primary synthesis. |
| Alfonso Arvez 2026 | Australia / VAED-PBS-NDI | Primary | Primary | Not pooled (k=2) | - | Possible partial overlap with Tan 2024. Overlap-removal sensitivity conducted. |
| Rim 2022 | United States / VA (non-active comparator) | - | - | Narrative (non-active comparator) | - | Non-active comparator; narrative only. |
| Jhu 2026 | United States / TriNetX federated EHR network (ESKD) | EHR-network stratum (Table S17); overlap sensitivity | EHR-network stratum (Table S17); overlap sensitivity | - | - | TriNetX overlap with Henney 2025. Retained as overlap-inclusive sensitivity evidence rather than pooled in the primary co-primary syntheses; restricted to end-stage kidney disease. Used only for qualitative triangulation of comparator and population dependence and not considered directly transportable to the broader T2DM population. |
| Chen 2026 | Global / TriNetX federated EHR network (peritoneal dialysis) | Narrative (non-active comparator) | Narrative (non-active comparator) | - | - | TriNetX overlap; non-user comparator, therefore outside the DPP-4i active-comparator framework and excluded from all pooled analyses and from the data-source stratification. Reported narratively. |
| Cheng 2026 | United States / TriNetX federated EHR network (psoriasis) | Not reported | Overlap sensitivity | - | - | TriNetX overlap with Henney 2025 and Jhu 2026. Eligible under the DPP-4i active-comparator framework but retained in the overlap-inclusive sepsis analysis because the source population was restricted to psoriasis. The separately matched GLP-1 receptor-agonist comparison was used only for qualitative triangulation of comparator dependence; neither contrast was considered directly transportable to the broader T2DM population. |
| Hsiao 2026 | United States / TriNetX federated EHR network (advanced chronic kidney disease) | Narrative (non-DPP-4i comparator) | Narrative (non-DPP-4i comparator) | - | - | TriNetX overlap. The comparator was a GLP-1 receptor agonist rather than a DPP-4 inhibitor, so the cohort remained narrative. It provided qualitative triangulation of comparator dependence in advanced chronic kidney disease but was not considered directly transportable to the broader T2DM population. |

**Table S4. Data-source overlap matrix and endpoint-prioritization rationale.**

| **Data source family** | **Studies** | **Population/time-window information available from reports** | **Shared endpoint(s)** | **Potential overlap** | **Primary handling rationale** |
| --- | --- | --- | --- | --- | --- |
| Hong Kong CDARS | Wu MZ 2022; Au 2022; Ng 2023 | Territory-wide Hong Kong CDARS cohorts of adults with T2DM receiving SGLT2i or DPP-4i; reported time windows and cohort construction overlap at least partially across publications. | Pneumonia, sepsis, mortality, ICU admission | High for source population; endpoint-specific overlap variable and not quantifiable from aggregate publications | Wu MZ 2022 was prioritized for incident pneumonia and incident sepsis because it directly reported the primary severe infection endpoints in a new-user active-comparator design. Au 2022 was retained as pneumonia sensitivity/narrative evidence. Ng 2023 was retained for ICU and mortality narrative evidence and for a sepsis substitution sensitivity analysis. |
| Australia linked data | Tan 2024; Alfonso Arvez 2026 | Australian linked pharmaceutical and hospital datasets; both used DPP-4i active comparators but addressed different hospitalization frameworks and analytic questions. | Sepsis (infection-related hospitalization reported by Alfonso Arvez 2026 only) | Partial possible overlap; exact individual-level overlap not reported | Both were retained for endpoint-specific analyses when they contributed distinct estimands. Overlap-removal sensitivity analyses excluding either Australian study preserved the direction and magnitude of the pooled estimates. Tan 2024 did not report overall infection-related hospitalization. |
| Taiwan NHIRD | Hu 2024; Wu CY 2026 | Taiwan national claims data; Hu used a non-active comparator framework, whereas Wu CY 2026 used a DPP-4i active-comparator new-user design. | Sepsis/septic shock | Possible source overlap, but target trial contrast differed materially | Wu CY 2026 was prioritized for the primary sepsis synthesis because it matched the DPP-4i active-comparator target contrast. Hu 2024 was retained as non-active-comparator narrative evidence only. |
| United States Veterans data | Rim 2022; Narasaki 2024 | Both used US Veterans data environments, but the comparator structure, exposure definition, and endpoint contribution differed. | Infection hospitalization or ICU infection outcomes | Possible broad health-system overlap; endpoint and comparator overlap limited | Narasaki 2024 was prioritized for infection-related hospitalization because it used incident-user comparative analyses with DPP-4i and GLP-1 RA comparators. Rim 2022 was retained as non-active-comparator narrative evidence. |
| No identified overlapping source | Brunetti 2021; Jeong 2023; Maki 2025; Ulambayar 2025 | United Kingdom CPRD-HES, South Korea HIRA, Japan DPC and Hungarian registry studies used distinct data environments. | Variable by endpoint | No direct data-source overlap identified | Included according to endpoint eligibility and comparator hierarchy. Henney 2025 was retained despite contributing materially to pneumonia heterogeneity because it captured a broader multinational EHR environment relevant to cross-setting applicability. |
| TriNetX federated EHR network | Henney 2025; Jhu 2026; Chen 2026; Cheng 2026; Hsiao 2026 | Federated EHR network cohorts differed in population and comparator: unselected T2DM (Henney), ESKD (Jhu), peritoneal dialysis (Chen), psoriasis (Cheng) and advanced CKD (Hsiao). Henney, Jhu and Cheng used DPP-4i active comparators; Chen used non-users and Hsiao used GLP-1 receptor agonists. | Incident pneumonia; incident sepsis | High overlap in network infrastructure; patient-level overlap cannot be quantified from aggregate publications and was not imputed. | Henney 2025 was retained in the primary pools. Jhu 2026 entered overlap-inclusive analyses for both co-primary outcomes; Cheng 2026 entered the sepsis overlap-inclusive analysis. Chen 2026 and Hsiao 2026 remained narrative because their comparators did not match the DPP-4i quantitative framework. All selected-population cohorts were used for sensitivity analysis or qualitative triangulation only and were not treated as directly transportable to the broader T2DM population. |

Transportability note: cohorts restricted to psoriasis, end-stage kidney disease, peritoneal dialysis or advanced chronic kidney disease were clinically selected populations. Their role was sensitivity analysis or qualitative triangulation of comparator dependence, not direct estimation for the broader T2DM population.

CDARS, Clinical Data Analysis and Reporting System; DPP-4i, dipeptidyl peptidase-4 inhibitor; EHR, electronic health record; GLP-1 RA, glucagon-like peptide-1 receptor agonist; ICU, intensive care unit; NHIRD, National Health Insurance Research Database; SGLT2i, sodium-glucose cotransporter 2 inhibitor; T2DM, type 2 diabetes mellitus.

# Section S4. ROBINS-I Risk of Bias - Domain-Level Assessment

Risk of bias was assessed independently by two reviewers across seven ROBINS-I domains, with disagreements resolved by consensus. Domains 3 to 5 were rated Low for most studies because active-comparator new-user designs minimise immortal-time and prevalent-user biases, and administrative databases rarely have large proportions of missing outcome data. Principal concerns were domain 1 (unmeasured confounding by frailty, vaccination, kidney function, prior infection and cardiopulmonary reserve) and domain 2 (selection bias in non-active-comparator designs and, for Henney 2025, a delayed index date). No study was rated overall Low risk of bias. Large sample size or propensity-score adjustment alone was not treated as sufficient for a Low rating.

**Table S5. ROBINS-I domain-level risk of bias.**

| **Study** | **D1 Confounding** | **D2 Selection** | **D3 Classification** | **D4 Deviations** | **D5 Missing data** | **D6 Outcome measurement** | **D7 Reporting** | **Overall** |
| --- | --- | --- | --- | --- | --- | --- | --- | --- |
| Brunetti 2021 | Moderate | Moderate | Low | Low | Low | Low | Low | Moderate |
| Wu MZ 2022 | Moderate | Moderate | Low | Low | Low | Low | Low | Moderate |
| Au 2022 | Moderate | Moderate | Low | Low | Low | Low | Low | Moderate |
| Rim 2022 | Serious | Moderate | Low | Low | Low | Low | Moderate | Serious |
| Jeong 2023 | Moderate | Low | Low | Low | Low | Low | Low | Moderate |
| Ng 2023 | Moderate | Moderate | Low | Low | Low | Low | Low | Moderate |
| Narasaki 2024 | Moderate | Low | Low | Low | Low | Low | Low | Moderate |
| Tan 2024 | Moderate | Low | Low | Low | Low | Low | Low | Moderate |
| Hu 2024 | Serious | Moderate | Low | Low | Low | Low | Moderate | Serious |
| Henney 2025 | Moderate | Moderate | Low | Low | Low | Low | Low | Moderate |
| Maki 2025 | Moderate | Moderate | Low | Low | Low | Low | Low | Moderate |
| Ulambayar 2025 | Serious | Moderate | Low | Low | Low | Moderate | Moderate | Serious |
| Wu CY 2026 | Moderate | Low | Low | Low | Low | Low | Low | Moderate |
| Alfonso Arvez 2026 | Moderate | Low | Low | Low | Low | Low | Low | Moderate |
| Jhu 2026 | Moderate | Low | Low | Low | Low | Low | Low | Moderate |
| Chen 2026 | Serious | Moderate | Low | Low | Low | Low | Moderate | Serious |
| Cheng 2026 | Moderate | Low | Low | Low | Low | Moderate | Low | Moderate |
| Hsiao 2026 | Moderate | Low | Low | Low | Low | Moderate | Moderate | Moderate |

D1, confounding; D2, selection of participants; D3, classification of interventions; D4, deviations from intended interventions; D5, missing data; D6, measurement of outcomes; D7, selection of the reported result.

# Section S5. ROBINS-I Risk of Bias - Overall Ratings with Rationale

**Table S6. ROBINS-I overall risk of bias and rationale.**

| **Study** | **Overall ROBINS-I** | **Main rationale** |
| --- | --- | --- |
| Brunetti 2021 | Moderate | Time-dependent exposure model and DPP-4i active comparator reduce immortal-time bias; residual confounding by frailty, vaccination, BMI, and cardiopulmonary reserve remains possible in CPRD. |
| Wu MZ 2022 | Moderate | New-user active-comparator design with propensity matching; CDARS overlap and residual confounding by kidney function and frailty remain concerns. |
| Au 2022 | Moderate | Prevalent new-user design with propensity matching; CDARS overlap and residual confounding remain. |
| Rim 2022 | Serious | Non-user comparator and drug-class-level exposure; confounding by indication and treatment-selection bias more likely than in active-comparator designs. |
| Jeong 2023 | Moderate | Nationwide active-comparator new-user cohort; residual confounding and outcome coding variability (respiratory composite) remain possible. |
| Ng 2023 | Moderate | Active-comparator cohort; critical-illness outcomes may reflect hospital-system thresholds; CDARS overlap interpreted cautiously. |
| Narasaki 2024 | Moderate | Incident-user cohort with multivariable and propensity-score analyses; residual confounding by CKD, frailty, and prior infection history possible. |
| Tan 2024 | Moderate | Active-comparator new-user outcome-wide cohort; multiple cause-specific coding and possible Australian population overlap require caution. |
| Hu 2024 | Serious | Non-SGLT2i comparator (mixed OAD/insulin); non-active-comparator structure and residual confounding substantially increase selection bias. |
| Henney 2025 | Moderate | Large multinational EHR active-comparator design; residual confounding and heterogeneity across TriNetX network sites remain possible. The index date was set 2 years after drug initiation, restricting the cohort to patients who survived and remained under observation for 2 years (selection domain rated moderate). |
| Maki 2025 | Moderate | Hospitalized pneumonia prognosis design; confounding by pneumonia severity, treatment selection, and institution type remain possible. |
| Ulambayar 2025 | Serious | Multiple drug classes pooled as comparator; not a clean SGLT2i versus DPP-4i active-comparator target-trial contrast; registry-based confounding. |
| Wu CY 2026 | Moderate | Active-comparator 1:2 matched design using NHIRD; residual confounding and administrative sepsis code definitions remain possible. |
| Alfonso Arvez 2026 | Moderate | Active-comparator post-discharge cohort with IPTW; hospitalization coding thresholds and Australian population overlap require caution. |
| Jhu 2026 | Moderate | Target-trial emulation with a DPP-4i active comparator and propensity-score matching within TriNetX; residual confounding, cross-institutional coding variability and restriction to end-stage kidney disease (with a high competing risk of death) limit interpretation. |
| Cheng 2026 | Moderate | Target-trial emulation with a DPP-4i active comparator, 12-month washout and exclusion of prevalent events within TriNetX; confounding by psoriasis severity and by systemic immunosuppressive therapy is incompletely addressed (no PASI or body-surface-area data), and severe sepsis is EHR-coded without adjudication. |
| Hsiao 2026 | Moderate | New-user active-comparator design with propensity-score matching and a 3-month lag within TriNetX; the comparator was a GLP-1 receptor agonist. Infection outcomes were EHR-coded without microbiological confirmation, and the reported sepsis HR, CI and P value appear internally discordant; the sepsis result is therefore described qualitatively. |
| Chen 2026 | Serious | Target-trial emulation in peritoneal dialysis using a non-user comparator; the absence of an active comparator makes confounding by indication and treatment-selection bias substantially more likely, as for other non-active-comparator cohorts in this review. |

No study was rated overall Low risk of bias because all analyses were observational and clinically important confounders (frailty, vaccination status, prior infection, kidney function, and cardiopulmonary reserve) were incompletely captured across data sources.

# Section S6. Sensitivity Analyses

Prespecified sensitivity analyses used the same REML random-effects model and modified Hartung-Knapp inference as the primary analyses. They included sequential leave-one-out analyses, substitution or removal of potentially overlapping data sources and overlap-inclusive analyses that added clinically selected cohorts from the same federated network. Prediction intervals were reported when at least three studies contributed. Incident sepsis remained a prespecified co-primary outcome; because substantial heterogeneity persisted and the primary 95% prediction interval crossed 1.0, pooled sepsis summaries are interpreted as exploratory.

**Table S7. Incident pneumonia sensitivity analyses.**

| **Analysis** | **Studies included** | **k** | **Estimate (95% CI)** | **I², %** |
| --- | --- | --- | --- | --- |
| Primary (REML-HK) | Brunetti, Wu MZ, Jeong, Henney, Alfonso Arvez 2026 | 5 | 0.65 (0.55 to 0.75) | 89.8 |
| Leave out Brunetti 2021 | Wu MZ, Jeong, Henney, Alfonso Arvez 2026 | 4 | 0.65 (0.55 to 0.78) | 92.0 |
| Leave out Wu MZ 2022 | Brunetti, Jeong, Henney, Alfonso Arvez 2026 | 4 | 0.65 (0.52 to 0.81) | 91.7 |
| Leave out Jeong 2023 | Brunetti, Wu MZ, Henney, Alfonso Arvez 2026 | 4 | 0.65 (0.53 to 0.81) | 87.6 |
| Leave out Henney 2025 | Brunetti, Wu MZ, Jeong, Alfonso Arvez 2026 | 4 | 0.61 (0.56 to 0.67) | 0.0 |
| Leave out Alfonso Arvez 2026 | Brunetti, Wu MZ, Jeong, Henney | 4 | 0.65 (0.53 to 0.81) | 89.0 |
| Substitute Au 2022 for Wu MZ 2022 | Brunetti, Au, Jeong, Henney, Alfonso Arvez 2026 | 5 | 0.66 (0.57 to 0.77) | 88.9 |
| Overlap-inclusive: add Jhu 2026 | Brunetti, Wu MZ, Jeong, Henney, Alfonso Arvez 2026, Jhu | 6 | 0.68 (0.55 to 0.85) | 93.1 |

**Table S8. Incident sepsis sensitivity analyses (prespecified co-primary outcome; pooled synthesis interpreted as exploratory).**

| **Analysis** | **Studies included** | **k** | **Estimate (95% CI)** | **I², %** |
| --- | --- | --- | --- | --- |
| Primary (REML-HK) | Wu MZ, Tan, Henney, Wu CY, Alfonso Arvez 2026 | 5 | 0.65 (0.53 to 0.80) | 90.6 |
| Leave out Wu MZ 2022 | Tan, Henney, Wu CY, Alfonso Arvez 2026 | 4 | 0.68 (0.55 to 0.85) | 88.6 |
| Leave out Tan 2024 | Wu MZ, Henney, Wu CY, Alfonso Arvez 2026 | 4 | 0.66 (0.49 to 0.88) | 91.9 |
| Leave out Henney 2025 | Wu MZ, Tan, Wu CY, Alfonso Arvez 2026 | 4 | 0.62 (0.48 to 0.82) | 92.4 |
| Leave out Wu CY 2026 | Wu MZ, Tan, Henney, Alfonso Arvez 2026 | 4 | 0.62 (0.48 to 0.80) | 92.2 |
| Leave out Alfonso Arvez 2026 | Wu MZ, Tan, Henney, Wu CY | 4 | 0.66 (0.49 to 0.89) | 88.1 |
| Overlap-inclusive: add Jhu 2026 | Wu MZ, Tan, Henney, Wu CY, Alfonso Arvez 2026, Jhu | 6 | 0.68 (0.56 to 0.83) | 91.0 |
| Overlap-inclusive: add Jhu 2026 and Cheng 2026 | Wu MZ, Tan, Henney, Wu CY, Alfonso Arvez 2026, Jhu, Cheng | 7 | 0.68 (0.58 to 0.81) | 89.4 |
| Substitute Ng 2023 (ICU admission for sepsis) for Wu MZ 2022 | Ng, Tan, Henney, Wu CY, Alfonso Arvez 2026 | 5 | 0.68 (0.57 to 0.81) | 85.5 |

**Table S9. Infection-related hospitalisation: contributing estimates and contextual evidence.**

| **Analysis** | **Studies included** | **k** | **Estimate (95% CI)** | **I², %** |
| --- | --- | --- | --- | --- |
| Contributing studies (not pooled; fewer than three) | Narasaki 2024 (HR); Alfonso Arvez 2026 (wIRR) | 2 | 0.74 (0.67 to 0.81); 0.74 (0.71 to 0.77) | - |
| Contextual estimate (not eligible for pooling) | Tan 2024, ICD-10-AM chapter A | 1 | 0.68 (0.63 to 0.74) | — |

Pneumonia and sepsis pooled associations remained directionally consistent in sensitivity analyses. Omitting Henney 2025 from the pneumonia pool reduced I² to 0.0% and yielded 0.61 (95% CI 0.56-0.67; prediction interval 0.54-0.69). Other pneumonia omissions left I² between 87.6% and 92.0%. For sepsis, I² remained 88.1%-92.4% after every omission, and substituting Ng 2023 for Wu MZ 2022 gave 0.68 (0.57-0.81; I²=85.5%; prediction interval 0.44-1.05). The overlap-inclusive analyses changed both co-primary means from approximately 0.65 to 0.68 while leaving prediction intervals across the null. Infection-related hospitalization: Tan 2024 did not report overall infection-related hospitalization. The original submission included its ICD-10-AM chapter J (respiratory diseases) estimate (IRR 0.71, 95% CI 0.66-0.75) in error. Its closest infection category, chapter A (A00-A99, primary diagnosis; IRR 0.68, 0.63-0.74), excludes respiratory, urinary and skin infections and is reported here only as contextual evidence and was not quantitatively combined with the two eligible overall infection-related hospitalisation estimates. I² was calculated from Cochran's Q.

**Table S10. Effect measure, model and event structure of studies contributing to the principal analyses.**

| **Study** | **Analyses** | **Measure** | **Model** | **Event structure** |
| --- | --- | --- | --- | --- |
| Brunetti 2021 | Pneumonia | HR | Time-dependent Cox (current use) | Time to first hospitalisation for community-acquired pneumonia |
| Wu MZ 2022 | Pneumonia; sepsis | HR | Cox, 1:2 propensity-score matched | Time to first event |
| Jeong 2023 | Pneumonia | HR | Cox, 1:1 propensity-score matched, as-treated | Time to first event |
| Henney 2025 | Pneumonia; sepsis | HR | Cox, 1:1 propensity-score matched, intention-to-treat | Time to first event within 12 months |
| Wu CY 2026 | Sepsis | HR | Cox, 1:2 matched | Time to first event |
| Narasaki 2024 | Infection-related hospitalisation (not pooled) | HR | Multivariable Cox | Time to first infection-related hospitalisation |
| Tan 2024 | Sepsis | IRR | Negative binomial | Rate of hospitalisations with unspecified sepsis (ICD-10-AM A41.9) as primary diagnosis; repeat admissions contribute |
| Alfonso Arvez 2026 | Pneumonia; sepsis; infection-related hospitalisation (not pooled) | wIRR | Negative binomial with IPTW | Rate of hospitalisations with the infection coded in any diagnosis field; repeat admissions contribute; follow-up to June 2018 (mean 1.6 years SGLT2i, 2.0 years DPP-4i) |

HR, hazard ratio; IRR, incidence rate ratio; IPTW, inverse probability of treatment weighting; wIRR, weighted incidence rate ratio.

**Table S11. Analyses stratified by effect measure (REML, modified Hartung-Knapp).**

| **Outcome** | **Stratum** | **k** | **Estimate (95% CI)** | **95% PI** | **I², %** | **P, subgroup difference** |
| --- | --- | --- | --- | --- | --- | --- |
| Incident pneumonia | HR only | 4 | 0.65 (0.53 to 0.81) | 0.37 to 1.16 | 89.0 | 0.40 |
|  | IRR only | 1 | 0.61 (0.55 to 0.66)a | - | - |  |
| Incident sepsis | HR only | 3 | 0.68 (0.40 to 1.15) | Not informativeb | 89.4 | 0.33 |
|  | IRR only | 2 | 0.60 (0.34 to 1.05)c | - | 0.0 |  |
| Infection-related hospitalisation | HR only | 1 | 0.74 (0.67 to 0.81)a | - | - | Not tested |
|  | IRR only | 1 | 0.74 (0.71 to 0.77)a | - | - |  |

a Single study, not pooled (pneumonia: Alfonso Arvez 2026; infection-related hospitalisation: Narasaki 2024 and Alfonso Arvez 2026). b With three studies the prediction interval uses 1 degree of freedom and is uninformative (0.03 to 13.48). c With two studies the modified Hartung-Knapp interval uses a t distribution with 1 degree of freedom; the conventional random-effects interval was 0.55 to 0.65. Subgroup differences were tested with a random-effects Q test (1 df) and had little power. I² was calculated from Cochran's Q. PI, prediction interval.

**Table S12. Study weights, influence diagnostics and complete leave-one-out results (REML, modified Hartung-Knapp).**

| **Outcome / study** | **Weight, %** | **Standardised residual** | **Cook's distance** | **Omitted: estimate (95% CI)** | **Omitted: 95% PI** | **Omitted: I², %** | **Omitted: τ²** |
| --- | --- | --- | --- | --- | --- | --- | --- |
| Incident pneumonia (τ² 0.010; 95% CI 0.002 to 0.170) |  |  |  |  |  |  |  |
| Brunetti 2021 | 3.5 | -1.03 | 0.04 | 0.65 (0.55 to 0.78) | 0.40 to 1.07 | 92.0 | 0.010 |
| Wu MZ 2022 | 19.8 | -0.16 | 0.00 | 0.65 (0.52 to 0.81) | 0.36 to 1.17 | 91.7 | 0.014 |
| Jeong 2023 | 24.7 | -0.51 | 0.07 | 0.65 (0.53 to 0.81) | 0.37 to 1.16 | 87.6 | 0.013 |
| Henney 2025 | 28.1 | +6.19 | 0.94 | 0.61 (0.56 to 0.67) | 0.54 to 0.69 | 0.0 | <0.001 |
| Alfonso Arvez 2026 | 23.9 | -0.50 | 0.06 | 0.65 (0.53 to 0.81) | 0.37 to 1.16 | 89.0 | 0.013 |
| Incident sepsis - exploratory pooled synthesis (τ² 0.024; 95% CI 0.007 to 0.225) |  |  |  |  |  |  |  |
| Wu MZ 2022 | 17.3 | -1.64 | 0.47 | 0.68 (0.55 to 0.85) | 0.37 to 1.27 | 88.6 | 0.016 |
| Tan 2024 | 17.3 | -0.45 | 0.04 | 0.66 (0.49 to 0.88) | 0.28 to 1.53 | 91.9 | 0.030 |
| Henney 2025 | 22.6 | +1.02 | 0.32 | 0.62 (0.48 to 0.82) | 0.29 to 1.35 | 92.4 | 0.025 |
| Wu CY 2026 | 22.3 | +1.28 | 0.39 | 0.62 (0.48 to 0.80) | 0.30 to 1.27 | 92.2 | 0.021 |
| Alfonso Arvez 2026 | 20.5 | -0.49 | 0.06 | 0.66 (0.49 to 0.89) | 0.28 to 1.55 | 88.1 | 0.030 |

Standardised residuals are externally standardised (each study compared with the model fitted without it). τ² confidence intervals are Q-profile intervals. I² was calculated from Cochran's Q. Infection-related hospitalisation was not pooled (Table S9). PI, prediction interval.

# Section S7. REML-Modified Hartung-Knapp Primary Estimates and Prediction Intervals

Primary inference used restricted maximum likelihood (REML) random-effects models with the modified Knapp-Hartung (mKH) adjustment. Let SE_HK denote the Hartung-Knapp standard error and SE_RE the conventional random-effects standard error from the same REML model. The corrected standard error was SE_mKH = max(SE_HK, SE_RE), and the confidence interval on the ratio scale was exp[mu_hat ± t_(k-1,0.975) x SE_mKH]. This is equivalent to the ad hoc safeguard implemented by test="adhoc" in *metafor*: the Hartung-Knapp adjustment is retained, but it cannot produce a standard error smaller than the unadjusted random-effects value. Prediction intervals used t(k-2) and incorporated τ² plus the adjusted mean-effect variance. They were reported only for k≥3 and are especially uncertain with few studies. Methodological reference: Röver C, Knapp G, Friede T. Hartung-Knapp-Sidik-Jonkman approach and its modification for random-effects meta-analysis with few studies. BMC Med Res Methodol. 2015;15:99.

Table S13. REML-modified Hartung-Knapp estimates and 95% prediction intervals.

| **Outcome** | **k** | **REML-mKH (95% CI)** | **DerSimonian-Laird (95% CI)** | **95% prediction interval** | **I²** | τ² (Q-profile 95% CI) |
| --- | --- | --- | --- | --- | --- | --- |
| Incident pneumonia | 5 | 0.65 (0.55 to 0.75) | 0.64 (0.56 to 0.73) | 0.45 to 0.93 | 89.8% | 0.010 (0.002 to 0.170) |
| Incident sepsis - exploratory pooled synthesis | 5 | 0.65 (0.53 to 0.80) | 0.66 (0.59 to 0.73) | 0.38 to 1.13a | 90.6% | 0.024 (0.007 to 0.225) |
| Infection-related hospitalization | 2 | Not pooled (k<3) | - | - | - | - |
| Incident pneumonia, overlap-inclusive sensitivity | 6 | 0.68 (0.55 to 0.85) | - | 0.39 to 1.21a | 93.1% | 0.035 |
| Incident sepsis, overlap-inclusive sensitivity | 7 | 0.68 (0.58 to 0.81) | - | 0.43 to 1.08a | 89.4% | 0.027 |

a Prediction interval crosses 1.0.

# Section S8. GRADE Certainty of Evidence Profile

Certainty of evidence was assessed using the GRADE approach. Because all included studies were observational, the initial certainty was low. Certainty was rated down further for serious inconsistency, imprecision, or residual confounding not adequately addressed by propensity methods.

**Table S14. GRADE certainty of evidence.**

| **Outcome** | **Certainty** | **Main reasons for rating** | **Interpretation** |
| --- | --- | --- | --- |
| Incident pneumonia | Very low | Observational evidence; serious inconsistency (I²=89.8%); residual confounding by frailty, vaccination, kidney function, prior infection, and cardiopulmonary reserve plausible. | Average association favoured SGLT2i; certainty is very low and substantial heterogeneity limits confidence across settings. |
| Incident sepsis (prespecified co-primary; pooled synthesis exploratory) | Very low | Observational evidence; serious inconsistency (I²=90.6%); prediction interval crossed 1.0; residual confounding remains plausible. | Average association favoured SGLT2i; certainty is very low and the prediction interval includes no association. |
| Infection-related hospitalization | Low | Observational evidence; two studies with concordant estimates (HR 0.74 and weighted IRR 0.74), not pooled because fewer than three studies were available; event structures also differed; hospitalization thresholds, coding practices and residual confounding remain possible. | Directionally favourable association in two cohorts; not causal evidence. |

# Section S9. Reporting-Guideline Checklists

Completed PRISMA 2020 and MOOSE checklists are provided as separate submission files and are not duplicated here. PRISMA 2020: Page MJ, McKenzie JE, Bossuyt PM, et al. BMJ 2021;372:n71. MOOSE: Stroup DF, Berlin JA, Morton SC, et al. JAMA 2000;283:2008-2012.

# Section S10. Exploratory Publication-Bias Assessment

Because no quantitative endpoint included 10 or more contributing studies, funnel plots and Egger regression tests were exploratory and cannot support firm conclusions about small-study effects or publication bias. Egger tests were reported only for the two five-study incident outcomes. The nominal sepsis P value (P=0.040) is presented for transparency but is not interpretable with only five studies and should not be used to infer publication bias. Infection-related hospitalisation was reported from two studies without pooling and is not shown in the funnel plots. Mortality outcomes were summarised narratively and were not included in small-study-effect assessments.

**Table S15. Exploratory Egger regression tests for small-study effects.**

| **Outcome** | **Studies, n** | **Egger test** | **P value** | **Interpretation** |
| --- | --- | --- | --- | --- |
| Incident pneumonia | 5 | Exploratory Egger regression | 0.092 | No firm inference; underpowered with fewer than 10 studies. |
| Incident sepsis | 5 | Exploratory Egger regression | 0.040 | Nominal asymmetry result; not interpretable with only five contributing studies. |
| Infection-related hospitalization | 2 | Not computed | NA | Not pooled (two studies). |

**Supplementary Fig. S1. Exploratory funnel plots.**

Each plot displays study-level log(effect estimate) on the x-axis and standard error on the y-axis; the vertical line indicates the pooled estimate and dashed curves indicate approximate pseudo-95% funnel bounds. Colored symbols represent individual study estimates, with symbol-study mappings shown within each panel. For black-and-white viewing, the symbols also differ by shape. These plots are provided for transparency only and should not be used to rule in or rule out publication bias given the small number of studies per endpoint.

# Section S11. Structured Heterogeneity Mapping

Formal subgroup meta-regression was not performed because fewer than 10 studies contributed to each quantitative endpoint and data-source architecture is collinear with population, outcome definition and index period. This table provides qualitative heterogeneity mapping by endpoint, region, data-source classification, outcome definition and healthcare delivery context; the corresponding quantitative exploratory stratification is given in Table S17.

**Table S16. Structured heterogeneity and clinical characterization mapping for primary endpoints.**

| **Primary endpoint** | **Study** | **Region** | **Data-source classification and design** | **Outcome ascertainment / definition framework** | **Potential heterogeneity driver and clinical context** |
| --- | --- | --- | --- | --- | --- |
| Incident pneumonia | Brunetti 2021 | United Kingdom | Single-jurisdiction database: CPRD Gold linked to HES; population cohort with time-dependent exposure | Hospitalization for community-acquired pneumonia based on linked primary-care and hospital records | Hospitalization-based pneumonia capture; admission threshold and linkage between primary-care and hospital data may differ from national insurance claims systems. |
| Incident pneumonia | Wu MZ 2022 | Hong Kong | Single-jurisdiction database: CDARS territory-wide EHR; new-user active-comparator cohort with propensity-score matching | Pneumonia identified within a hospital-linked EHR/administrative system | CDARS source-population overlap with Au 2022 and Ng 2023; prioritized for primary pneumonia and sepsis pools because it provided endpoint-specific active-comparator incident estimates. |
| Incident pneumonia | Jeong 2023 | South Korea | Single-jurisdiction database: HIRA national claims; active-comparator new-user cohort with propensity-score matching | Pneumonia within a broader adverse respiratory event framework using national claims diagnoses | Universal insurance system and claims-based coding may produce different ascertainment thresholds than EHR network data. |
| Incident pneumonia | Henney 2025 | Multinational | Multinational federated EHR network: TriNetX; active-comparator new-user cohort (index date 2 years after initiation) with propensity-score matching | Incident pneumonia identified from multi-institutional EHR-coded records | Principal driver of pneumonia heterogeneity in leave-one-out analysis; multi-country and multi-institutional EHR variation may enrich tertiary-care phenotypes and heterogeneous coding practices. |
| Incident pneumonia | Alfonso Arvez 2026 | Australia | Single-jurisdiction database: VAED-PBS-NDI linked pharmaceutical and hospital data; active-comparator post-discharge cohort with IPTW | Infection-specific hospitalization including pneumonia after SGLT2i or DPP-4i initiation | Hospital-admission outcome and post-discharge design may reflect care transitions and regional admission thresholds; possible partial overlap with Australian PBS-linked studies. |
| Incident sepsis | Wu MZ 2022 | Hong Kong | Single-jurisdiction database: CDARS territory-wide EHR; new-user active-comparator cohort with propensity-score matching | Sepsis identified within a hospital-linked EHR/administrative system | Territory-wide hospital coding and CDARS overlap; sepsis ascertainment may depend on local diagnostic coding and blood-culture/severity documentation. |
| Incident sepsis | Tan 2024 | Australia | Single-jurisdiction database: NDSS-PBS-hospital-NDI linked data; active-comparator new-user outcome-wide cohort | Cause-specific hospitalization including sepsis | Hospitalization threshold and cause-specific coding; possible partial source-population overlap with Alfonso Arvez 2026, addressed in overlap-removal sensitivity analysis. |
| Incident sepsis | Henney 2025 | Multinational | Multinational federated EHR network: TriNetX; active-comparator new-user cohort (index date 2 years after initiation) with propensity-score matching | Severe sepsis identified from EHR-coded records | EHR network heterogeneity across institutions and countries; potential variation in clinical documentation, coding intensity, and severity mix. |
| Incident sepsis | Wu CY 2026 | Taiwan | Single-jurisdiction database: NHIRD national claims; active-comparator 1:2 matched cohort | Sepsis and septic shock using administrative claims definitions | Reimbursement-based ICD coding and national insurance thresholds; different NHIRD time window from Hu 2024 and treated as non-overlapping for primary analysis. |
| Incident sepsis | Alfonso Arvez 2026 | Australia | Single-jurisdiction database: VAED-PBS-NDI linked pharmaceutical and hospital data; active-comparator post-discharge cohort with IPTW | Infection-specific hospitalization including sepsis after SGLT2i or DPP-4i initiation | Post-discharge cohort context and admission-based endpoint; possible partial overlap with Tan 2024, addressed in sensitivity analysis. |
| Incident pneumonia | Jhu 2026 | United States (TriNetX) | Multinational federated EHR network: TriNetX, restricted to end-stage kidney disease; target-trial emulation, active-comparator new-user, PS-matched | Incident pneumonia identified from multi-institutional EHR-coded records | Federated EHR architecture plus severe population selection. The near-null estimate (0.95) lies outside the single-country prediction interval, but competing mortality in end-stage kidney disease is an equally credible explanation; architecture and population cannot be separated with two EHR cohorts. |
| Incident sepsis | Jhu 2026 | United States (TriNetX) | Multinational federated EHR network: TriNetX, restricted to end-stage kidney disease; target-trial emulation, active-comparator new-user, PS-matched | Sepsis identified from multi-institutional EHR-coded records | As above. Sepsis coding in end-stage kidney disease may be affected by dialysis-access infection, frequent healthcare contact and competing mortality. |
| Incident sepsis | Cheng 2026 | United States (TriNetX) | Multinational federated EHR network: TriNetX, restricted to psoriasis with comorbid type 2 diabetes; target-trial emulation, active-comparator new-user, PS-matched | Severe sepsis identified from multi-institutional EHR-coded records | Federated EHR architecture plus population selection restricted to psoriasis. A separately matched GLP-1 receptor-agonist comparison was null, providing qualitative triangulation of comparator dependence but not a directly transportable estimate for the broader T2DM population. |

**Section S12. Exploratory Post Hoc Data-Source Stratification**

This assessment was devised after the primary analyses, was not specified at registration and is reported as exploratory and hypothesis-generating. Contributing cohorts were classified as either single-jurisdiction database cohorts or cohorts drawn from a multinational federated EHR network. Strata were pooled only when at least three sufficiently independent and clinically comparable cohorts were available. Federated-network strata were not pooled because patient-level overlap could not be quantified and the cohorts represented clinically selected populations. Formal meta-regression was not performed because fewer than 10 studies contributed to each endpoint and data-source architecture is collinear with population, outcome definition and index period. The table provides study-level detail for the exploratory result summarised briefly in the main text.

**Table S17. Exploratory post hoc stratification of the co-primary endpoints by data-source architecture.**

| **Endpoint / stratum** | **Contributing cohorts (point estimate)** | **Pooled estimate (95% CI)** | **I², %** | **95% prediction interval** |
| --- | --- | --- | --- | --- |
| Incident pneumonia - single-jurisdiction databases | Brunetti 2021 (0.48); Wu MZ 2022 (0.63); Jeong 2023 (0.61); Alfonso Arvez 2026 (0.61) | 0.61 (0.56 to 0.67) | 0.0 | 0.54 to 0.69 |
| Incident pneumonia - multinational federated EHR network | Henney 2025 (0.75); Jhu 2026 (0.95) | Not pooled (k=2) | - | - |
| Incident sepsis - single-jurisdiction databases | Wu MZ 2022 (0.52); Tan 2024 (0.60); Wu CY 2026 (0.77); Alfonso Arvez 2026 (0.60) | 0.62 (0.48 to 0.82) | 92.4 | 0.29 to 1.35 |
| Incident sepsis - multinational federated EHR network | Henney 2025 (0.75); Jhu 2026 (0.87); Cheng 2026 (0.69) | Not pooled (k=3; overlapping selected cohorts) | - | - |

Among single-jurisdiction database cohorts, pneumonia estimates were concordant whereas sepsis estimates remained heterogeneous. The pneumonia stratum is numerically identical to the prespecified leave-one-out analysis omitting Henney 2025 and therefore provides no independent evidence for a data-architecture effect. Sepsis estimates from cohorts drawn from the multinational federated EHR network ranged from 0.69 to 0.87 and overlapped the single-jurisdiction database estimates. Although three federated-network sepsis cohorts were available, they were not pooled because patient-level overlap could not be quantified and two cohorts were restricted to clinically selected populations (end-stage kidney disease and psoriasis). Data-source architecture is confounded with population selection and outcome definition; these findings are hypothesis-generating only. CI, confidence interval; EHR, electronic health record; PI, prediction interval.

# Section S13. Cohorts Added by the Updated Search

Four eligible cohorts were identified in the final database search and incorporated according to the prespecified eligibility and endpoint-level overlap rules. Jhu 2026 and Cheng 2026 used DPP-4i active comparators but overlapped the retained broader federated-network cohort and were assigned to overlap-inclusive sensitivity analyses. Chen 2026 used a non-user comparator and Hsiao 2026 used a GLP-1 receptor agonist comparator; both remained narrative. Because all four cohorts involved clinically selected populations, their principal role was sensitivity analysis or qualitative triangulation rather than direct transportability to the broader T2DM population.

**Jhu 2026 (Sci Rep; TriNetX US Collaborative Network; end-stage kidney disease)**

Eligible: adults with type 2 diabetes, SGLT2i exposure, DPP-4i active comparator, adjusted hazard ratios with 95% confidence intervals for sepsis and pneumonia in a real-world cohort. Overlaps the retained multinational cohort from the same federated network (Henney 2025). Under the primary overlap rule the unselected multinational cohort was retained and Jhu 2026 was carried into the overlap-inclusive sensitivity analyses for both co-primary endpoints. ROBINS-I: moderate.

**Chen 2026 (Clin Kidney J; TriNetX; peritoneal dialysis)**

Eligible for narrative synthesis only. The comparator is a non-user group rather than a DPP-4 inhibitor, which places the study outside the quantitative active-comparator framework specified in the PROSPERO record. Retained in the narrative layer, not pooled. ROBINS-I: serious (non-active comparator).

**Cheng 2026 (J Am Acad Dermatol; TriNetX; psoriasis with comorbid type 2 diabetes)**

Eligible: 8,799 propensity-score-matched pairs of SGLT2i and DPP-4i initiators, reporting severe sepsis (0.689, 95% CI 0.594-0.799). Because the source population was restricted to psoriasis and overlapped the broader TriNetX cohort, Cheng 2026 was included in the overlap-inclusive sepsis analysis rather than the primary pool. The k=7 analysis yielded 0.68 (0.58-0.81), I²=89.4%, τ²=0.027 and prediction interval 0.43-1.08. A separately matched comparison against GLP-1 receptor agonists was null (1.026, 0.892-1.180) and was used only for qualitative triangulation of comparator dependence. Neither selected-population contrast was treated as directly transportable to the broader T2DM population. ROBINS-I: moderate.

**Hsiao 2026 (Diabetes Res Clin Pract; TriNetX; advanced chronic kidney disease)**

Eligible for narrative synthesis only because the comparator was a GLP-1 receptor agonist. In 22,393 propensity-score-matched pairs with advanced chronic kidney disease, pneumonia did not differ between GLP-1 receptor agonists and SGLT2i (0.98, 95% CI 0.92-1.04); sepsis was reported as not significantly different. The reported sepsis hazard ratio, confidence interval and P value appear internally discordant, so the result is described qualitatively rather than quoted numerically. This selected-population cohort was used only for qualitative triangulation and was not considered directly transportable to the broader T2DM population. ROBINS-I: moderate.

**Effect of the top-up search on the principal analyses**

None of the four studies altered the primary pneumonia, sepsis or infection-related-hospitalisation syntheses, which remain based on five and five DPP-4i active-comparator cohorts; infection-related hospitalisation is reported from two cohorts without pooling. Cheng 2026 expanded the sepsis overlap-inclusive sensitivity analysis from k=6 to k=7; the average remained approximately 0.68 and the prediction interval continued to cross the null. The principal contribution of the new cohorts is interpretive triangulation rather than a directly generalisable numerical estimate.

# Section S14. Design Comparison of the Federated-Network Pneumonia Cohort

**Table S18. Design of the multinational federated-network cohort compared with the four single-jurisdiction pneumonia cohorts.**

| **Cohort** | **Data source and setting** | **Population and time zero** | **Pneumonia definition** | **Follow-up** | **Adjustment; measure** |
| --- | --- | --- | --- | --- | --- |
| Brunetti 2021 | UK CPRD Gold linked to hospitalisation data (primary care and hospital) | Adults with T2DM; time-dependent current use of SGLT2i vs DPP-4i | Hospitalisation for community-acquired pneumonia | Mean 1.7 years | Time-dependent Cox; HR |
| Wu MZ 2022 | Hong Kong CDARS, territory-wide public-hospital EHR | New users 2015-2019; follow-up from initiation | Incident pneumonia in hospital-linked records | Median 2.3 years | 1:2 PSM, Cox; HR |
| Jeong 2023 | South Korea HIRA nationwide claims | New users 2016-2020; follow-up from initiation; as-treated | Pneumonia (reported separately within a respiratory composite); claims diagnoses | Median 0.66 years | 1:1 PSM, Cox; HR |
| Alfonso Arvez 2026 | Victoria, Australia; linked hospital, dispensing and death data | New users after hospital discharge; follow-up from first dispensing | Hospitalisation with pneumonia coded in any diagnosis field | Mean 1.6 years (SGLT2i) and 2.0 years (DPP-4i) | IPTW, negative binomial; wIRR |
| Henney 2025 | TriNetX Global Collaborative Network; multinational federated EHR, mainly secondary and tertiary care in North America and Western Europe | New users; index date 2 years after drug initiation; intention-to-treat | Incident pneumonia, ICD-10 J13-J18 in EHR | 12 months | 1:1 PSM (including BMI, HbA1c, eGFR), Cox; HR |

CDARS, Clinical Data Analysis and Reporting System; CPRD, Clinical Practice Research Datalink; EHR, electronic health record; HIRA, Health Insurance Review and Assessment Service; IPTW, inverse probability of treatment weighting; PSM, propensity-score matching.

# Abbreviations

ACNU, active-comparator new-user; CDARS, Clinical Data Analysis and Reporting System; CI, confidence interval; CENTRAL, Cochrane Central Register of Controlled Trials; CKD, chronic kidney disease; CPRD, Clinical Practice Research Datalink; DL, DerSimonian-Laird; DPC, Diagnosis Procedure Combination; DPP-4i, dipeptidyl peptidase-4 inhibitor; EHR, electronic health record; ESKD, end-stage kidney disease; GEE, generalised estimating equations; GLP-1 RA, glucagon-like peptide-1 receptor agonist; GRADE, Grading of Recommendations Assessment, Development and Evaluation; HES, Hospital Episode Statistics; HIRA, Health Insurance Review and Assessment Service; HK, Hartung-Knapp; mKH, modified Knapp-Hartung; HR, hazard ratio; ICU, intensive care unit; IPTW, inverse probability of treatment weighting; IRR, incidence rate ratio; NDI, National Death Index; NDSS, National Diabetes Services Scheme; NHIRD, National Health Insurance Research Database; PBS, Pharmaceutical Benefits Scheme; PD, peritoneal dialysis; PI, prediction interval; PRISMA, Preferred Reporting Items for Systematic Reviews and Meta-Analyses; PSM, propensity-score matching; REML, restricted maximum likelihood; ROBINS-I, Risk Of Bias In Non-randomized Studies of Interventions; SGLT2i, sodium-glucose cotransporter 2 inhibitor; T2DM, type 2 diabetes mellitus; VA, Veterans Affairs; VAED, Victorian Admitted Episodes Dataset; wIRR, weighted incidence rate ratio.
